# Integrative analysis of hepatic transcriptional profiles reveals genetic regulation of atherosclerosis in hyperlipidemic Diversity Outbred-F1 mice

**DOI:** 10.1038/s41598-023-35917-8

**Published:** 2023-06-10

**Authors:** Myungsuk Kim, M. Nazmul Huda, Levi W. Evans, Excel Que, Erik R. Gertz, Nobuyo Maeda-Smithies, Brian J. Bennett

**Affiliations:** 1 Department of Nutrition, University of California, Davis, CA USA; 2 Western Human Nutrition Research Center, Agricultural Research Service, US Department of Agriculture, Davis, CA USA; 3 Department of Pathology and Laboratory Medicine, University of North Carolina at Chapel Hill, Chapel Hill, NC USA; 4 Korea Institute of Science and Technology (KIST), Gangneung, Gangwon-Do Republic of Korea; 5 Division of Bio-Medical Science and Technology, KIST School, University of Science and Technology (UST), Seoul, 02792 Republic of Korea

**Keywords:** Genetics, Systems biology, Cardiology

## Abstract

Atherogenesis is an insipidus but precipitating process leading to serious consequences of many cardiovascular diseases (CVD). Numerous genetic loci contributing to atherosclerosis have been identified in human genome-wide association studies, but these studies have limitations in the ability to control environmental factors and to decipher cause/effect relationships. To assess the power of hyperlipidemic Diversity Outbred (DO) mice in facilitating quantitative trait loci (QTL) analysis of complex traits, we generated a high-resolution genetic panel of atherosclerosis susceptible (DO-F1) mouse cohort by crossing 200 DO females with C57BL/6J males carrying two human genes: encoding apolipoprotein E3-Leiden and cholesterol ester transfer protein. We examined atherosclerotic traits including plasma lipids and glucose in the 235 female and 226 male progeny before and after 16 weeks of a high-fat/cholesterol diet, and aortic plaque size at 24 weeks. We also assessed the liver transcriptome using RNA-sequencing. Our QTL mapping for atherosclerotic traits identified one previously reported female-specific QTL on Chr10 with a narrower interval of 22.73 to 30.80 Mb, and one novel male-specific QTL at 31.89 to 40.25 Mb on Chr19. Liver transcription levels of several genes within each QTL were highly correlated with the atherogenic traits. A majority of these candidates have already known atherogenic potential in humans and/or mice, but integrative QTL, eQTL, and correlation analyses further pointed *Ptprk* as a major candidate of the Chr10 QTL, while *Pten* and *Cyp2c67* of the Chr19 QTL in our DO-F1 cohort. Finally, through additional analyses of RNA-seq data we identified genetic regulation of hepatic transcription factors, including *Nr1h3,* contributes to atherogenesis in this cohort. Thus, an integrative approach using DO-F1 mice effectively validates the influence of genetic factors on atherosclerosis in DO mice and suggests an opportunity to discover therapeutics in the setting of hyperlipidemia.

## Introduction

Atherosclerosis, a major cause of coronary artery disease (CAD), is a highly complex disease caused by the interaction of genetic and environmental factors^1,2^. Early evidence for the genetic cause of atherosclerosis was based on the demonstration of familial aggregation and heritability estimates^3,4^, which initiated a search to identify risk alleles. The advent of genome-wide association studies (GWAS) has yielded an unbiased genome-wide approach that has identified novel atherosclerosis candidate genes. To date, GWAS have identified over 100 individual CAD susceptibility loci^5^. In aggregate, however, these loci only explain a fraction of the heritability of atherosclerosis and even less of the overall risk of disease^6^. Thus, the specific causes and determinants of atherosclerosis remain to be elucidated, including environmental factors, diet, epigenome, and molecular mechanisms^7,8^.

A complementary approach to studying the genetic factors of atherosclerosis in humans is to use forward genetic approaches in experimental model organisms where the environment can be monitored and closely controlled. These studies benefit from the ability to comprehensively phenotype the animals for clinical traits and ascertain tissues for molecular traits such as quantitation of RNA levels, allowing a precise assessment of the impact of genetic factors on each phenotype while minimizing confounding factors. Thus, quantitative trait locus (QTL) analysis has identified hundreds of genetic loci associated with various clinical traits including atherosclerosis^9^. Furthermore, genetic reference panels such as the Hybrid Mouse Diversity Panel (HMDP) and Diversity Outbred (DO) mice have made it possible to perform high-resolution mapping of complex traits with approximately 1 Mb of resolution^9–14^.

In this study, we investigated the genetic regulation of atherosclerosis and known risk factors in Diversity Outbred F1 (DO-F1). DO-F1 mice were generated from crossing Diversity Outbred (DO) females with inbred C57BL/6J male mice which harbored two transgenes: human cholesteryl ester transfer protein (CETP) and apolipoprotein E-Leiden (APOE-Leiden). Mice naturally lack functional CETP gene, and the CETP transgene reduces the concentration of high-density lipoprotein (HDL), and the APOE-Leiden transgene reduces the clearance of triglyceride-rich lipoproteins^15^. Thus, as compared to wild-type mice, the carriers of hyperlipidemia-inducing APOE-Leiden and CETP transgenes display a lipoprotein cholesterol profile similar to humans with familial hyperlipidemia type II or III^15^. We then fed the DO-F1 offspring with a high fat and high cholesterol (HFHC) diet and examined atherosclerotic traits including plasma lipids and glucose before and after 16 weeks of a high-fat/cholesterol diet.

To the best of our knowledge, this study reports the first example of quantitative trait loci (QTL) mapping of atherosclerosis in DO-F1 mice. We determine genetic effects of atherosclerotic traits and gene expression (eQTLs). By incorporating aortic lesion area QTLs with eQTLs, we identify multiple genetic effects on atherosclerosis that colocalize with *cis*-acting eQTLs. Finally, we investigate genetic regulation of hepatic transcription factors (TFs) associated with atherosclerotic traits and demonstrate how genetic variation of TFs influences downstream candidate genes and susceptibility to atherosclerosis.

## Results

### Characterizing atherosclerotic traits in DO-F1 mice

**Figure 1:**
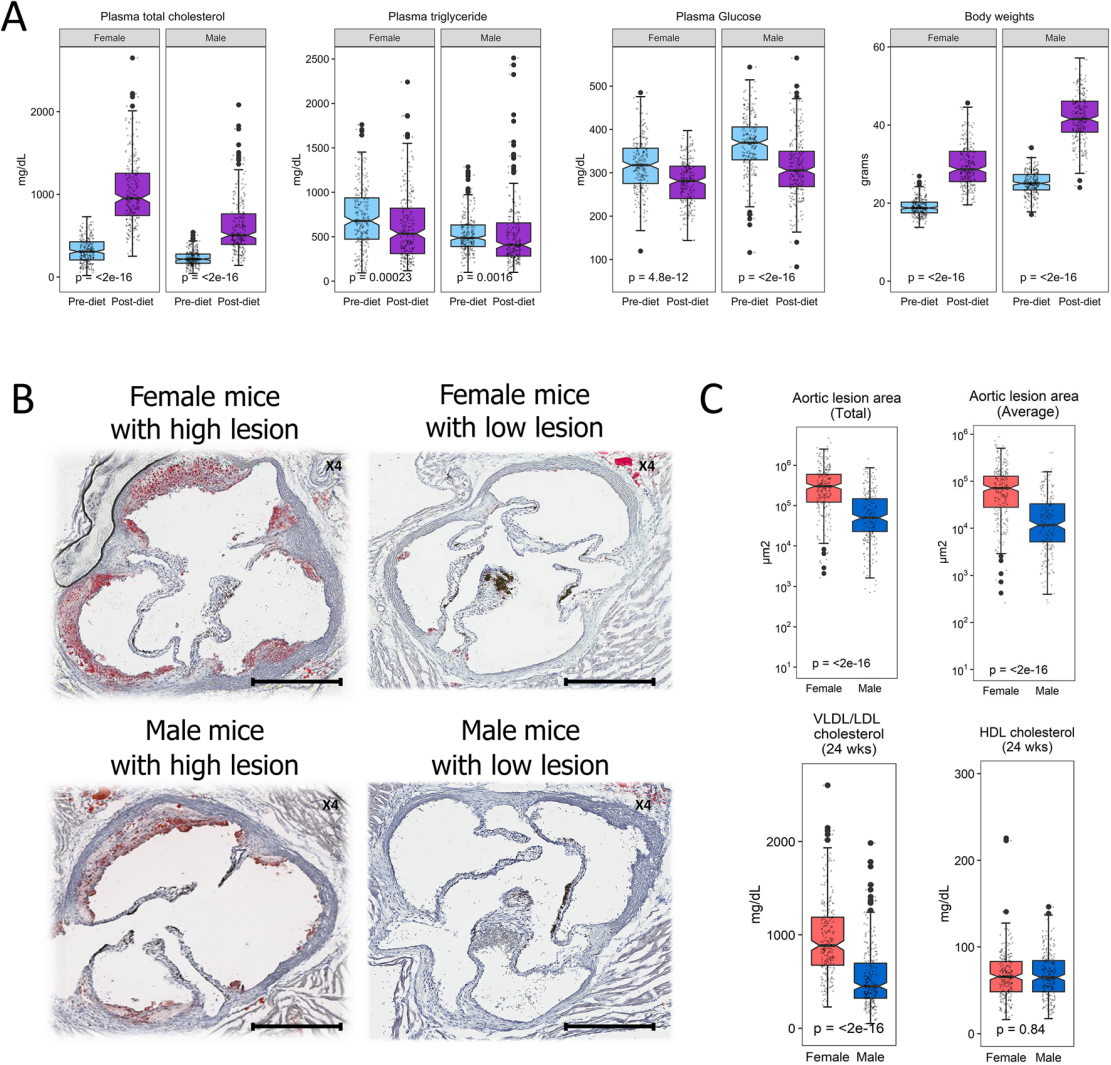
Sex differences and phenotypic variations in atherosclerotic traits in Diversity Outbred (DO)-F1 mice. A total of 238 female and 234 male (J:DO × CETP/ApoE3 Leiden) F1 mice were assessed for atherosclerotic traits. (**A**) Plasma total cholesterol, triglyceride, glucose, and body weight before and after high-cholesterol (HFHC) diet challenge for 16 weeks. The p-values were Wilcoxon signed-rank test between sexes for each atherosclerotic trait. (**B**) Oil red O stained cross section of aortic roots of representative females and males with either high or low lesion areas at 24 weeks. Scale bars, 400 μm. (**C**) Sex differences in aortic lesion areas and plasma very low-density lipoprotein cholesterol/low-density lipoprotein cholesterol (VLDL-C/LDL-C) and high density lipoprotein cholesterol (HDL).

An overview of the study design and research scheme is depicted in Fig. S1. Prior to the induction of severe hyperlipidemia with the HFHC diet (Table S1), we observed significant differences in atherosclerotic traits between sexes and tremendous variation among DO-F1 mice (Fig. 1). For example, mean plasma total cholesterol (TC) was 318 mg/dL in females at 8 weeks of age and 228 mg/dL in males but ranged from 18 to 729 mg/dL in females and 44.8 mg/dL and 540 mg/dL in males. This represents a tenfold phenotypic variance among the DO-F1 mice and a 1.4-fold difference between sexes. Similar variation within and between sexes was observed for triglyceride (TG), glucose, and body weight (Fig. 1A).

To induce hyperlipidemia, we fed male and female DO-F1 mice a HFHC diet for 16 weeks. The body weight increased 1.6-fold and 2.7-fold, respectively over the course of the diet treatment. As expected, the increase in body weight was accompanied by increased metabolic dysfunction as indicated by the severe increase in circulating total cholesterol (mean TC of 1033 mg/dL in females while 608 mg/dL in males). In both sexes, this represents a threefold increase in circulating lipids. Further assessment of the lipoprotein profiles indicated that the majority of the circulating cholesterol was contained in the very-low-density lipoprotein cholesterol/low-density lipoprotein cholesterol (VLDL/LDL) classes (Fig. 1). Although smaller in extent, diet effects on plasma glucose and triglycerides in individual mice were also observed (Fig. S2).

Following 16 weeks of diet, we assessed the development of atherosclerosis in the DO-F1 mice. Similar to the risk factors for atherosclerosis, there was tremendous variation in atherosclerotic lesion size among DO-F1 mice and between sexes. The mean aortic lesion area was 91,638 ± 83,287 μm^2^ and 22,211 ± 30,113 μm^2^, respectively in females and males (Fig. 1B,C, P < 2 × 10^–16^) indicating a significant effect of sex on atherosclerosis. These results are consistent with the eight DO founder strains-F1 mice that were used for the strain survey experiments (Fig. S3 and Table S2; P = 0.001). Here, the mean aortic lesion area in DO founder strains varied between 113,010 ± 97,437 μm^2^ in females and 24,416 ± 36,308 μm^2^ in males, a 4.8-fold difference of the mean size between the two sexes. To determine the relationship between aortic lesion area and atherosclerotic traits in the DO-F1, we performed Spearman correlation analysis in females and males separately (Table 1). Pre-diet, at 8 weeks of age, plasma TC and TG measured showed a significant correlation with the aortic lesion area regardless of sex. At 24 weeks, after 16 weeks of diet, plasma TC, VLDL-C/LDL-C, and TG were also positively correlated with the aortic lesion area in both sexes. On individual animal bases, all plasma traits showed strong correlations between the pre-diet and post-diet periods, regardless of sex (Table S3).

**Table 1:** Spearman correlations between aortic lesion area and atherosclerotic traits in DO-F1 mice.

Trait	Unit	Weeks	Male mice		Female mice	
			ρ	p	ρ	p
Body weight	g	8	0.011	0.87	− 0.005	0.94
Total cholesterol	mg/dL	8	0.24	2.8 × 10^–04^	0.20	0.002
Glucose	mg/dL	8	− 0.07	0.29	− 0.14	0.035
Triglyceride	mg/dL	8	0.24	2.9 × 10^–04^	0.20	0.002
Body weight	g	24	− 0.049	0.45	0.012	0.85
Total cholesterol	mg/dL	24	0.49	1.9 × 10^–15^	0.38	1.7 × 10^–09^
VLDL/LDL-cholesterol	mg/dL	24	0.50	5.4 × 10^–16^	0.38	1.4 × 10^–09^
HDL-cholesterol	mg/dL	24	−0.051	0.44	0.064	0.32
Glucose	mg/dL	24	− 0.058	0.38	0.11	0.08
Triglyceride	mg/dL	24	0.32	4.5 × 10^–07^	0.13	0.04

n = 472 (238 females and 234 males).

### Genetic regulation of atherosclerotic traits in hyperlipidemic DO-F1 mice

We first assessed the heritability for clinical traits to examine the genetic contribution to atherosclerotic traits. The heritability of traits ranged between 0.31–0.67 and was 0.5 for aortic lesion area indicating that similar to humans and other genetic approaches using mouse models, a proportion of the variation in these traits can be attributed to genetics (Table 2). Based on our observation of sex-specific differences in atherosclerotic traits, we conducted QTL analysis in a sex additive model, female mice, and male mice, respectively. Prior to the induction of severe hyperlipidemia by the HFHC diet feeding, we identified QTLs for plasma triglycerides, cholesterol, and glucose, all of which are associated with atherosclerosis risk (Table 3 and Table S4). For example, a significant association (P < 0.05) was identified for plasma glucose measured at 8 weeks on Chr 1 at 176 Mb in male mice. This QTL had a logarithms of odds ratios (LOD) score of 8.4, overlapped with the previously identified QTLs including body weight (Bw8q1), TC (Tcq11), HDL-C (Phdlc5), and triglyceride (Tgl5), and associated with *ApoA2 *locus^16–19^. A novel QTL for plasma TG was found on Chr 6 in males at 125 Mb with a LOD score 9.5. Following 16 weeks of high-fat feeding to induce hyperlipidemia, QTLs were identified for plasma TG on Chr 1 in females at 36.5 Mb and on Chr 6 in a sex additive model at approximately 125 Mb (Fig. S4 and Table S5). The Chr 6 locus overlaps with previously observed QTLs including atherosclerosis (*Ath37*), body mass index (*Bmiq4*), and plasma apolipoprotein B (*Pabr1*)^20–22^. We also performed QTL analysis of the changes in plasma traits in response to the HFHC. We identified several suggestive QTL, however, no additional significant QTLs were found (Table S6).

**Table 2:** Narrow sense heritability for atherosclerotic traits in DO-F1 mice.

Trait	Unit	Weeks	Narrow sense heritability		
			All mice	Female mice	Male mice
Body weight	g	8	0.67	0.93	0.76
		24	0.54	1.00	0.94
Total cholesterol	mg/dL	8	0.39	0.75	0.62
		24	0.37	0.42	0.77
VLDL/LDL-cholesterol	mg/dL	24	0.35	0.42	0.74
HDL-cholesterol	mg/dL	24	0.31	0.43	0.45
Glucose	mg/dL	8	0.34	0.40	0.43
		24	0.38	0.46	0.59
Triglyceride	mg/dL	8	0.33	0.63	0.58
		24	0.38	0.46	0.59
Aortic lesion area	µm^2^/section	24	0.50	0.64	0.98
Total aortic lesion area	µm^2^	24	0.46	0.62	1.00

n = 461 (235 females and 226 males).

**Table 3:** Significant QTLs for atherosclerotic traits in three models and strain difference in regression coefficient of the association between each trait and marker SNP.

Model	Traits	Weeks	Chr	Position (Mbp)	LOD	CI (low)^a^	CI (hi)^a^	LOD threshold (p < 0.63)	LOD threshold (p < 0.05)	#Genes	Marker
Female mice	Plasma triglyceride	24	1	36.50	8.11	35.81	38.31	6.58	8.08	31	UNC454349
Female mice	Aortic lesion area (Average)	24	4	132.73	6.51	55.60	134.86	6.44	7.60	502	UNC8247956
Female mice	Aortic lesion area (Average)	24	10	28.85	7.30	22.90	30.75	6.44	7.60	33	JAX00286290
Female mice	Aortic lesion area (Average)	24	14	68.04	6.74	37.40	70.35	6.44	7.60	237	UNC24171938
Male mice	Plasma glucose	8	1	176.42	8.42	175.48	179.92	6.50	7.79	22	JAX00012935
Male mice	Plasma triglyceride	8	6	125.69	9.53	122.57	126.02	6.61	7.97	61	UNCHS018944
Male mice	Plasma glucose	24	11	100.41	8.45	100.11	104.92	6.49	7.72	122	UNC20243587
Male mice	Aortic lesion area (Average)	24	19	38.17	7.92	32.00	40.23	6.43	7.56	58	UNCHS047709
Sex additive	Plasma triglyceride	24	6	124.86	7.78	122.58	125.54	6.39	7.64	70	UNC12014770
Sex additive	Aortic lesion area (Average)	24	19	33.00	6.55	32.09	39.09	6.37	7.69	54	UNCHS047622

n = 461 (235 females and 226 males).

Significance is considered only when QTL results were P < 0.05. For the aortic lesion area trait only, significant and suggestive QTL results are included in the table.

^a^95% Bayesian credible interval was calculated as implemented by the bayesint function in R/QTL2.

**Figure 2:**
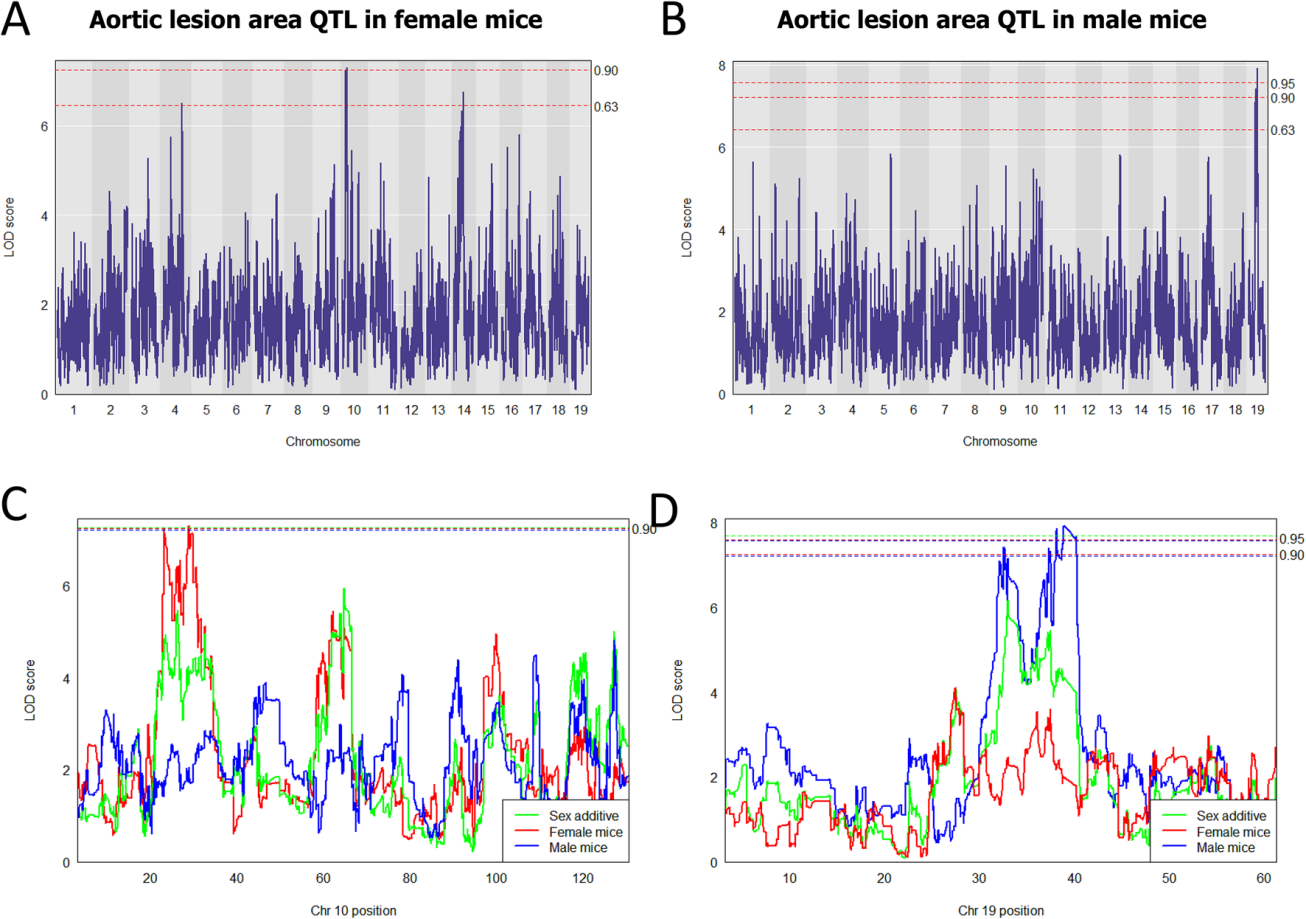
Aortic lesion area QTL of DO-F1 mice in three models. (**A**,**B**) Genome-wide aortic lesion area QTLs on Chr 10 in female mice (**A**) and Chr 19 in male mice (**B**) fed a synthetic HFHC diet for 16 weeks. Dashed lines correspond to P = 0.05 (0.95 significant), P < 0.1 (0.90 highly suggestive), or P < 0.63 (0.36 suggestive) thresholds. (**C**,**D**) Comparison of aortic lesion area QTLs in three models on Chr 10 in female mice (**C**) and Chr 19 in male mice (**D**). Green, sex additive model; red, female mice; blue, male mice. Dashed lines correspond to P < 0.05 (significant) and P < 0.1 (highly suggestive).

We identified two genome-wide significant loci associated with average atherosclerotic lesion size. A female-specific model identified one highly suggestive (P < 0.1) QTL on Chr 10 (Fig. 2A,C) which overlaps with the previously identified *Ath11* locus^23^. This QTL had a LOD 7.3, spanned 22.90 to 30.75 Mb on Chr 10, and contains 51 protein-coding genes (Table S7). One significant (P < 0.05) and novel QTL was identified on Chr 19 in a male-specific model (Fig. 2B,D). This complex QTL with a LOD 7.92 spans 32.09 to 39.09 Mb and contains 61 protein-coding genes (Table S7). The proximal boundary of this QTL indicates that mice harboring CAST and A/J allele have increased lesion size while the distal QTL boundary indicates that mice with 129 and PWK alleles have increased lesion size (Figs. 4C and 5C).

### Prioritization of positional candidate genes underlying atherosclerotic lesion size QTLs in hyperlipidemic DO-F1 Mice

To prioritize candidate genes at the loci associated with atherosclerosis, we chose to incorporate gene expression analysis in the livers of DO-F1 mice, with assumptions that (1) the liver is the most important organ for lipoprotein metabolism, and is the source of hyperlipidemia, and (2) regulation of gene expression in the livers would likely represent the expression of multiple cell types including endothelial cells, smooth muscle cells, macrophages and fibroblasts involved in the plaque development in aortic tissues. Our data showed the livers express 28 and 58 genes located in the confidence interval for the aortic lesion area QTLs on Chr 10 and 19, respectively (Table S6). On Chr10, *Rps12*, *Moxd1,* and *Ptprk* expressions were negatively correlated with aortic lesion in females only with adjusted *P*-values 0.033, 0.044, and 0.037 respectively, while *Enpp1* and *Echdc1* were negatively correlated only in males, both with adjusted *P*-values of 0.001. Involvement in atherogenesis of all these genes has been experimentally demonstrated in the literature except for *Echdc1,* which nevertheless is involved in lipid metabolism. Among the genes located within the QTL range on Chr 19, strong negative correlations (adjusted P < 0.01) between aortic lesion and the liver expression of *Tbpl1*, *Pten*, *Pank1*, *Rbp4,* and *Cyp2c67* were found only in males, while of *Minpp1*, *Atad1*, *Marchf5*, *Exo6* and *Hells* only in females. Again, many of these genes have been experimentally demonstrated/implicated to play roles in atherosclerosis.

**Figure 3:**
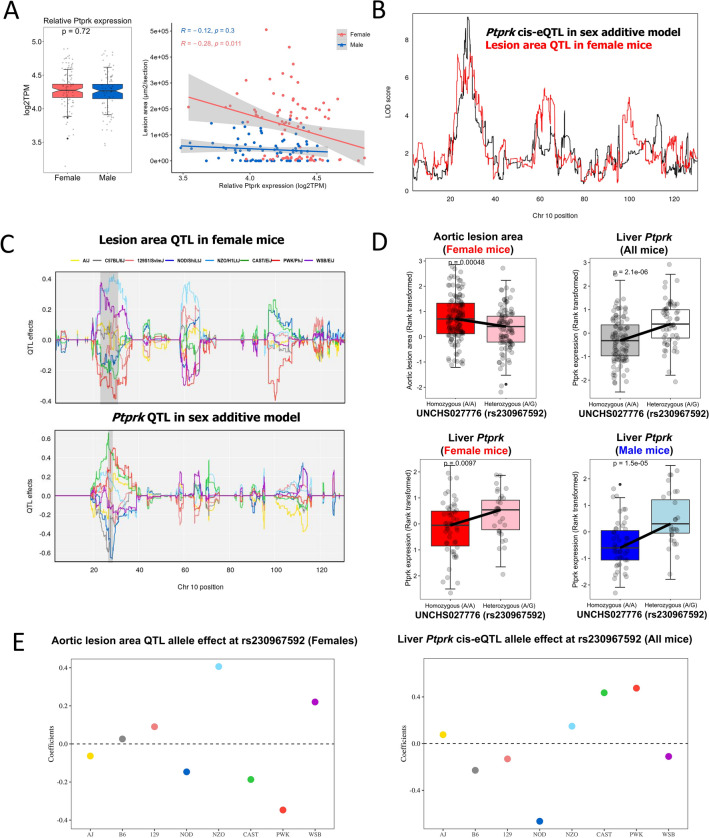
Co-localization of aortic lesion area QTL with liver gene expression of *Ptprk* in females. (**A**) *Ptprk* gene expression (log2TPM, transcripts per million) is negatively correlated with aortic lesion area in females (ρ = − 0.28, P < 0.011), not males (ρ = − 0.12, P < 0.3). (**B**) LOD score profiles of *Ptprk cis*-eQTL on Chr 10 in a sex additive model (black line) and of aortic lesion area QTL in female mice (red line). (**C**) Estimated founder allele effect plots for aortic lesion area QTL in females and *Ptprk cis*-eQTL in sex additive model. Female aortic lesion area was linked to the high allele in NZO and WSB strain and low allele in CAST and PWK strains and *Ptprk* gene expression was linked to the high allele in CAST and PWK strains and low allele in WSB strain at the Chr 10 QTL. (**D**) Associations of a SNP (rs230967592) in the *Ptprk* gene with aortic lesion area in females (red color) or liver *Ptprk* gene expression in all mice (grey color), females (red color), and males (blue color). (**E**) Estimated founder strain levels of aortic lesion area in females and liver *Ptprk* gene expression in all mice were inferred from the founder strain coefficients observed at the SNP (rs230967592).

We next focused on liver *cis*-eQTLs colocalizing with aortic lesion area QTLs. First, sequence read and alignment quality was assessed by RNA-sequencing of total RNA isolated from liver tissue (Table S8). Next, *cis* and *trans*-eQTL from the sex additive at the Chr 10 and 19 loci are presented in Table S9. We found 10 *cis*-eQTLs that colocalized with female aortic lesion area QTL on Chr 10, and 16 *cis*-eQTLs that colocalized with male aortic lesion area QTL on Chr 19 (Table S10). We further prioritized these positional candidate genes by calculating the correlation between these *cis*-eQTLs genes and aortic lesion area. For example, of the ten genes that have *cis*-eQTLs in the Chr 10 QTL interval, *Moxd1* (monooxygenase DBH Like 1) and *Ptprk* (protein tyrosine phosphatase receptor type K transporter) genes were significantly correlated with aortic lesion area in females (Fig. 3A and Table S10) and *Ptprk cis*-eQTLwas associated with the same locus as aortic lesion area (Fig. 3B). We next compared whether shared founder allele effects were observed between aortic lesion areas QTL in females and *Ptprk cis*-QTL. The CAST and PWK alleles on Chr 10 QTL in females were associated with smaller lesion size (Fig. 3C). However, we observed that *Ptprk* mRNA levels were higher in mice harboring CAST and PWK alleles than non-carriers at this locus (Fig. 3C). Additionally, single nucleotide polymorphism (SNPs) within the *Ptprk* gene were associated with both aortic lesion area (P < 0.001) and *Ptprk* gene expression (P < 0.001 in all mice, females, or males) (Fig. 3D,E and Table 4). These data suggest that genetic variants at approximately 28.1–28.5 Mb on Chr 10 are associated with decreased hepatic *Ptprk* gene expression level and increased aortic lesion area.

**Table 4:** List of SNPs in candidate genes that showed significant difference of aortic lesion area and gene expression between heterozygous and homozygous genotypes.

Gene	Maker	Chr	Position	SNP	Strains that have minor alleles	Major allele (Allele from B6)	Minor allele
*Ptprk*	UNCHS027770	10	28.142762	rs29316154	CAST;PWK	C	T
*Ptprk*	UNC17651750	10	28.218847	rs243273871	CAST;PWK	C	A
*Ptprk*	JAX00016183	10	28.288723	rs46801305	CAST	T	G
*Ptprk*	UNC17653128	10	28.315729	rs248898676	CAST;PWK	T	G
*Ptprk*	UNC17653533	10	28.348571	rs52604325	CAST;PWK	G	T
*Ptprk*	UNCHS027772	10	28.390664	rs36371657	CAST;PWK	A	G
*Ptprk*	UNCHS027775	10	28.403443	rs50071536	CAST;PWK;WSB	C	T
*Ptprk*	UNCHS027776	10	28.405695	rs230967592	CAST;PWK	A	G
*Ptprk*	UNCHS027779	10	28.463659	rs238966765	PWK	C	G
*Ptprk*	UNC17655660	10	28.485286	rs225638435	CAST;PWK	C	T
*Pten*	UNC30214131	19	32.802285	rs30401869	B6;129;CAST;PWK	T	C
*Cyp2c67*	UNCHS047733	19	39.942755	rs223861560	PWK	C	T

**Figure 4:**
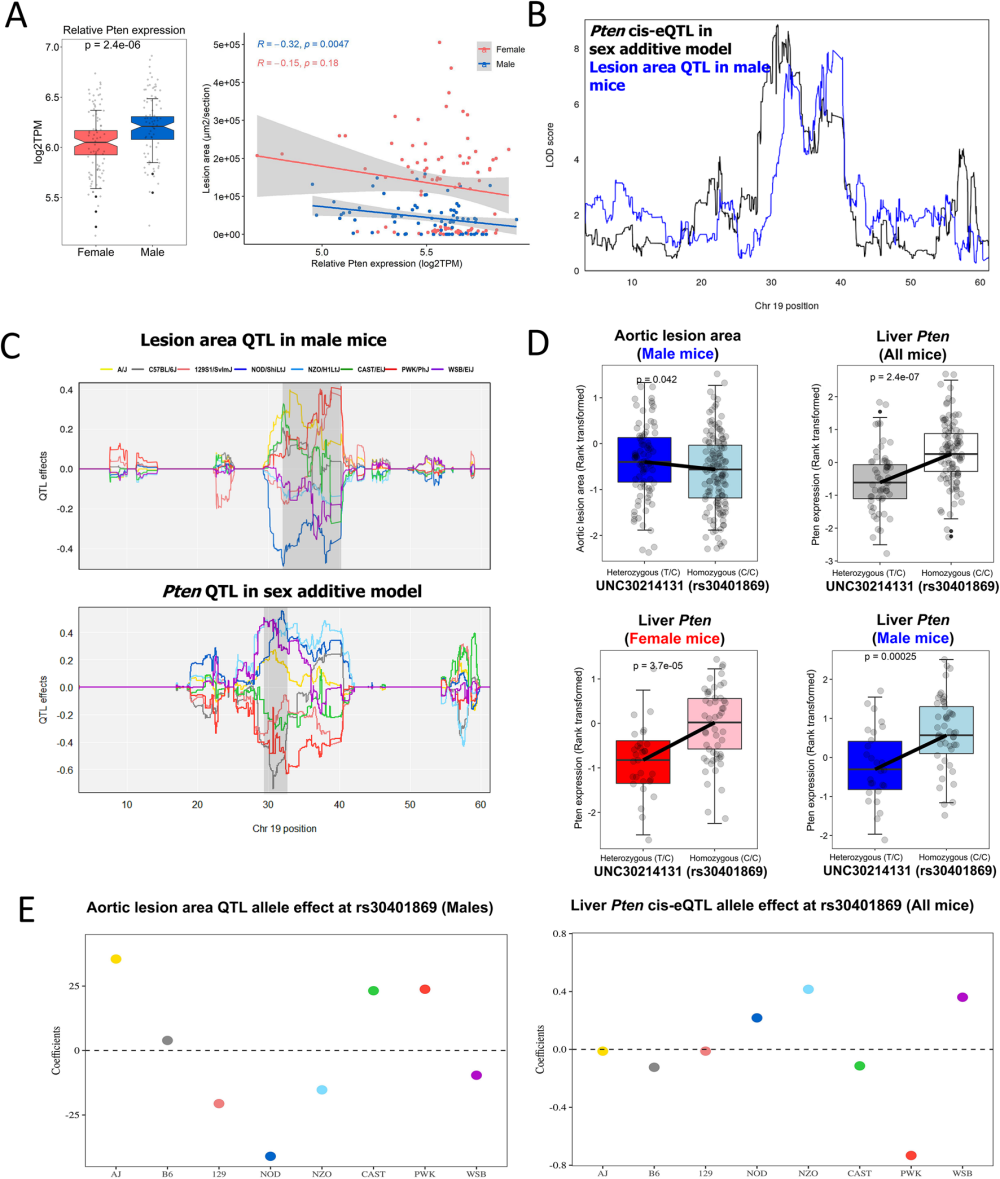
Co-localization of aortic lesion area QTLs with liver gene expression of *Pten* in males. (**A**) *Pten* gene expression is higher in males than in females (P < 1 × 10^–5^) and negatively correlated with aortic lesion area in males (ρ = − 0.32, P < 0.0047), not females (ρ = − 0.15, P < 0.18). (**B**) LOD profiles on Chr 19 highlight a significant association between the liver *Pten* gene expression and aortic lesion area in male mice. A black line represents the LOD score for a *cis*-eQTL of *Pten* expression in a sex additive model, and a blue line for aortic lesion area QTL in male mice. (**C**) Estimated founder allele effect plots for aortic lesion area QTL in males and *Pten cis*-eQTL in sex additive model. Male aortic lesion area was linked to the high allele in PWK strain and low allele in NOD strain and *Pten* gene expression was linked to the high allele in NOD strain and low allele in PWK strain at the Chr 19 QTL. (**D**) Association between a SNP (rs30401869) in the *Pten* gene and aortic lesion area in males (blue color) or liver *Pten* gene expression in all mice (grey color), females (red color), and males (blue color). (**E**) Estimated founder strain levels of aortic lesion area in males and liver *Pten* gene expression in all mice were inferred from the founder strain coefficients observed at the SNP (rs30401869).

**Figure 5:**
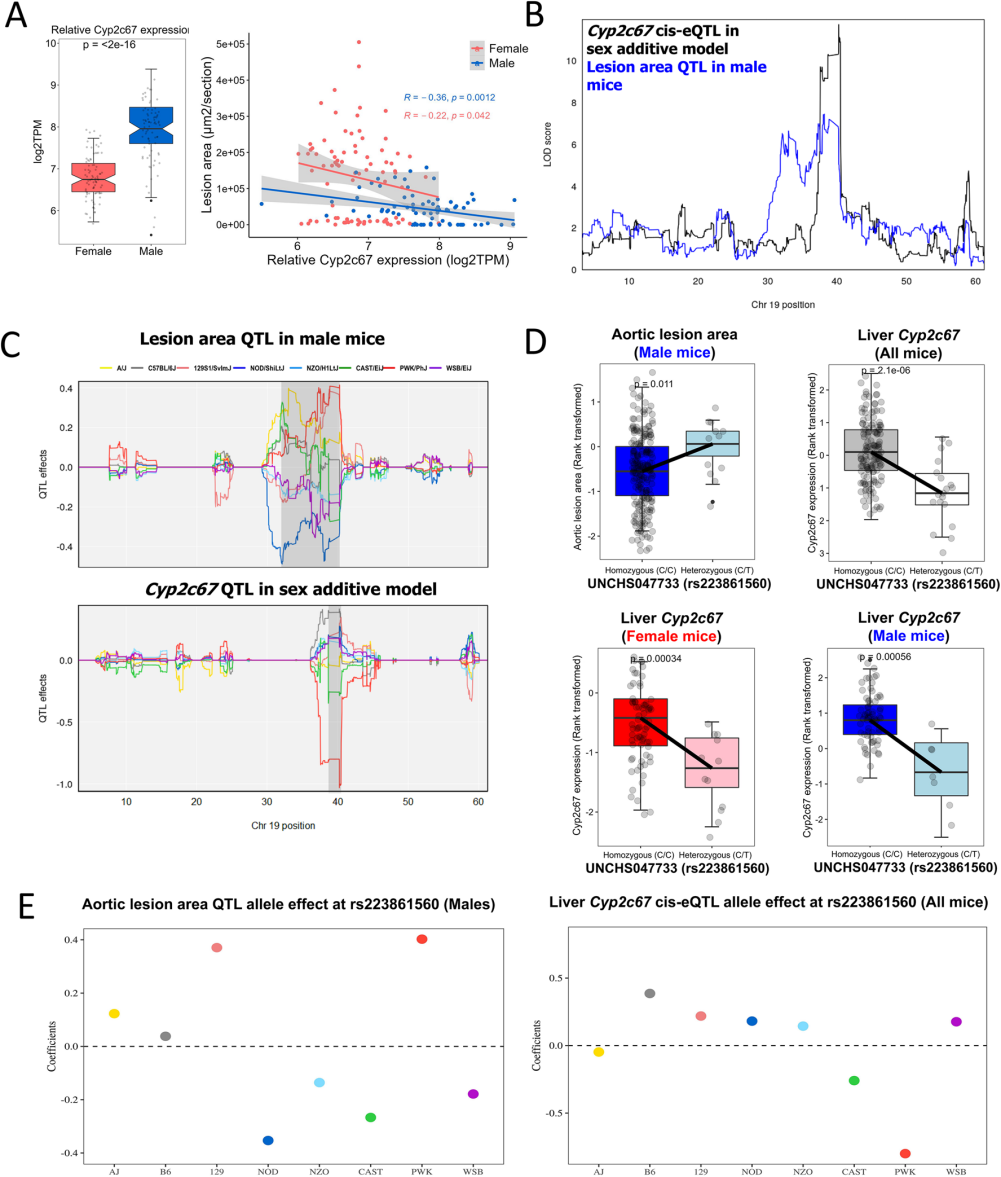
Aortic lesion area QTLs co-localized with liver gene expression of *Cyp2c67* in males. (**A**) *Cyp2c67* gene expression (log2TPM) is higher in males than in females (P < 2 × 10^–16^) and negatively correlated with aortic lesion area in males (ρ = − 0.36, P < 0.0012) and females (ρ = − 0.22, P < 0.042). (**B**) LOD profiles on Chr 19 highlighting a locus with significant association between the liver *Cyp2c67* gene expression and aortic lesion area in male mice. A black line represents the LOD score for *Cyp2c67*, indicating a *cis*-eQTL in a sex additive model, and a blue line for aortic lesion area QTL in male mice. (**C**) Estimated founder allele effect plots for aortic lesion area QTL in males and *Cyp2c67 cis*-eQTL in sex additive model. Male aortic lesion area was linked to the high allele in PWK strain and *Cyp2c67* gene expression was linked to the low allele in PWK strain at the Chr 19 QTL. (**D**) Association between a SNP (rs223861560) in the *Cyp2c67* gene and aortic lesion area in males (blue color) or liver *Cyp2c67* gene expression in all mice (grey color), females (red color), and males (blue color). (**E**) Estimated founder strain levels of aortic lesion area in males and liver *Cyp2c67* gene expression in all mice were inferred from the founder strain coefficients observed at the SNP (rs223861560).

Similarly, of 16 genes that have *cis*-eQTLs at the Chr 19 locus, *Pten* (phosphatase and tensin homolog) and *Cyp2c67* (cytochrome P450, family 2, subfamily c, polypeptide 67) were negatively correlated (P = 0.005) with aortic lesion area in males (Figs. 4A and 5A) and are physically located within the atherosclerosis QTL interval on Chr 19 (Figs. 4B and 5B). Our assessment of the shared founder allele in males for aortic lesion area QTL and the *Pten* or *Cyp2c67 cis*-eQTL revealed a complexity of this atherosclerosis QTL interval on Chr 19. Namely, the allelic effects at the proximal and distal boundaries of the QTL interval were different in the atherosclerosis QTL interval on Chr 19 (Figs. 4C and 5C). At the proximal boundary, the A/J, CAST, and PWK alleles were associated with larger lesion size, and the NOD, NZO, and WSB alleles were associated with smaller lesions (Figs. 4C and 5C). At the distal boundary, we found that the 129 and PWK alleles were associated with larger lesion size, and the NOD, CAST, NZO, and WSB alleles were associated with smaller lesions (Figs. 4C and 5C). In common in both boundaries on Chr 19, the PWK allele was associated with larger lesion size, and the NOD, NZO, and WSB alleles were associated with smaller lesions. The *cis*-eQTL for *Pten* and *Cyp2c67* were associated with PWK alleles but showed the opposite allele effect pattern to the aortic lesion area QTL (Figs. 4C and 5C). SNPs within the genomic coordinates of both *Pten* and *Cyp2c67* were associated with both aortic lesion area (P < 0.05) (Figs. 4D and 5D and Table 4) and their respective mRNA levels (P < 0.001) (Figs. 4E and 5E and Table 4). The direction of these associations suggests that SNPs present in PWK led to a decrease of *Pten* and *Cyp2c67* expression and an increase of aortic lesion size at the colocalized locus in males. Although we used only a limited number of DO-F1 mice for RNA-seq analysis, we attempted to prioritize genes at the Chr 19 locus using mediation analysis^24^. This preliminary analysis supports that expression of *Pten* could be a strong candidate mediator of atherosclerosis (Table S11). In contrast, analysis did not support the role of *Cyp2c67* as a mediator gene*.* Taken together, the expression of multiple genes within Chr10 and Chr19 QTL regions are negatively associated with atherosclerosis, multiple alleles may be acting additively within the QTL, and the genetic associations in the DO-F1 population influence atherosclerosis in a sex-dependent manner.

### Identification of liver transcription factors with CVD GWAS, genetic regulation, and correlation with atherosclerosis

A panel of DO-F1 mice extensively characterized for their atherosclerotic traits, genetic variants, and liver transcriptome could provide a powerful resource for uncovering the mechanistic basis for the disease. To validate this concept, we next applied our integrated approaches to our hypothesis that differences in the expression of TFs can be critical determinants of atherosclerosis susceptibility which has been supported in the literature^25^. To evaluate whether expression of the liver transcriptome and genetic regulation are useful for dissecting the molecular basis of liver TF functions for atherosclerosis, we examined the expression and genetic regulation of liver TFs correlating with atherosclerotic traits in DO-F1 mice and investigated whether they were associated with CVD-related traits in human GWAS. First, we prioritized TF binding sites (TFBSs) of mouse TFs that were identified by large-scale chromatin immunoprecipitation sequencing analysis^26^ in promoter regions (i.e., 2 kb upstream of the transcription start site). Of 453 mouse TF listed in the HOCOMOCO database v11, we limited our analysis to the 265 TFs expressed in liver of DO-F1 mice.

**Figure 6:**
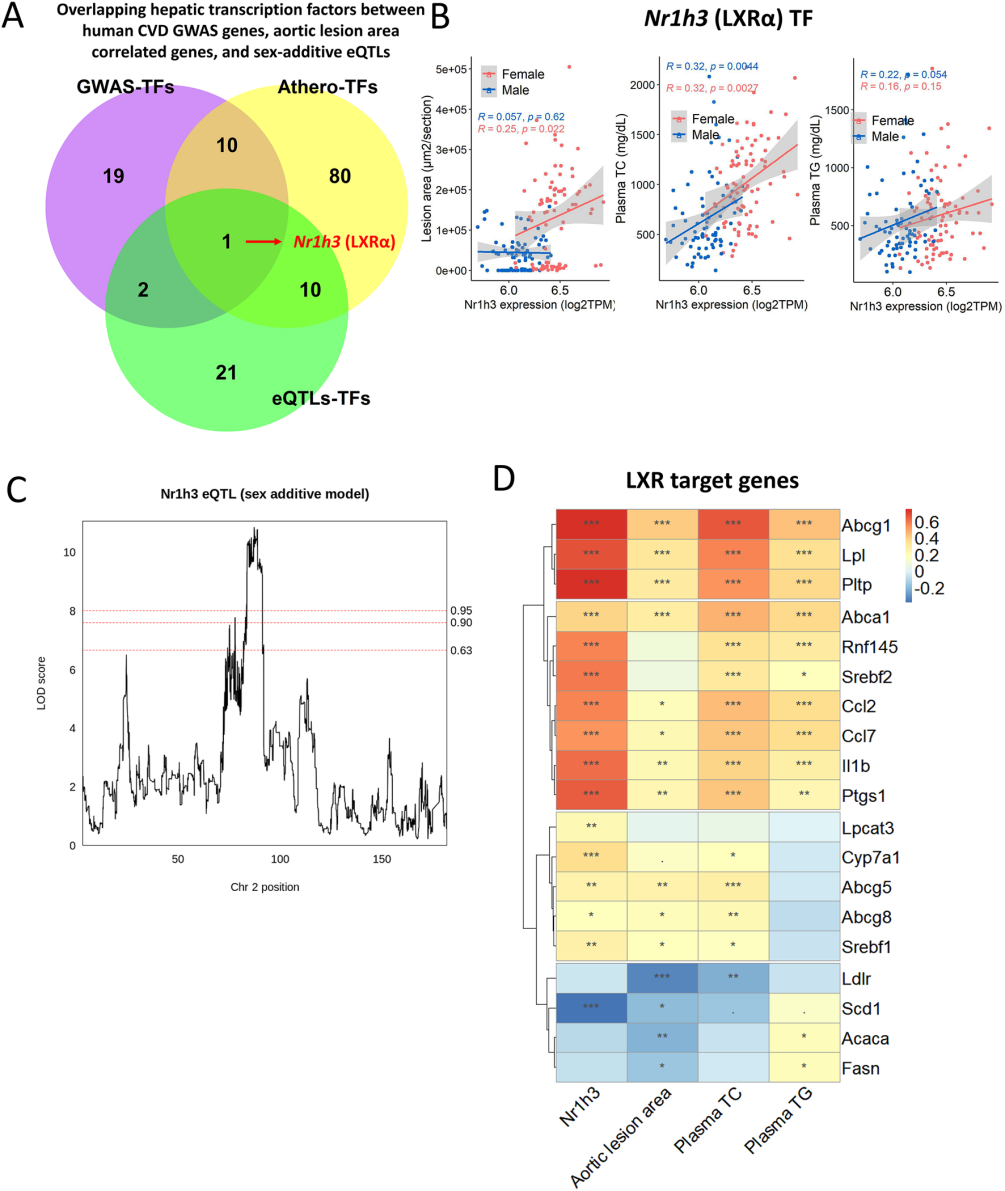
Association of a hepatic TF, *Nr1h3,* with atherosclerosis in the DO-F1 mice. (**A**) Identification of *Nr1h3* as a candidate hepatic TFs associated with atherosclerosis traits. Among TFs listed in the HocomocoV11, 265 are expressed in the DO-F1 livers. Among them, 32 TFs (purple circle) are associated with atherosclerosis-related traits in human GWAS, 34 TFs (green circle) have eQTLs in a sex additive model and yellow circle contains 101 TFs correlated with the aortic lesion area (adjusted P < 0.05) in all DO-F1 mice. Only *Nr1h3* satisfies all three criteria. (**B**) *Nr1h3* gene expression is positively correlated with aortic lesion area in females (ρ = 0.25, P < 0.05), not males (ρ = 0.057, P > 0.05), and positively correlated with plasma total cholesterol in both sexes (ρ = 0.32, P < 0.001) by Spearman correlation. (**C**) LOD profiles of the liver *Nr1h3* gene expression QTL in a sex additive model. *Nr1h3* is located at 91 Mb on Chr 2. Dashed lines correspond to P < 0.05 (significant), P < 0.1 (highly suggestive) or P < 0.63 (suggestive) thresholds. (**D**) Spearman correlation between liver *Nr1h3* gene expression, aortic lesion area and plasma total cholesterol and triglyceride with target genes of *Nr1h3* TF. The p-values were adjusted using the BH FDR procedure. “***” P < 0.001, “**” P < 0.01, and “*” P < 0.05.

We next sought to identify liver TFs that are genetically regulated and contribute to the susceptibility of atherosclerosis. We focused on liver TFs with significant eQTLs, correlated with atherosclerosis, and associated with CVD-related traits in GWAS. Of the 34 liver TFs with eQTLs, nine showed significant correlations with aortic lesion area, plasma TC, and plasma TG (Table 5). Of these nine TFs that were associated with atherosclerosis, only *Nr1h3* (encoding Liver X receptor alpha) was mechanistically associated with plasma lipids and atherosclerosis in GWAS. (Fig. 6A). For example, *Nr1h3* expression was positively correlated with aortic lesion area in females and with plasma TC in both sexes (Fig. 6B). In addition, *Nr1h3* had a *cis*-eQTL in a sex additive model (Fig. 6C).

**Table 5:** Spearman correlation between aortic lesion area and 34 transcription factors with significant eQTL in sex additive model.

Gene	Chr	Start (Mbp)	End (Mbp)	eQTLs	Aortic lesion area		Total cholesterol		Triglyceride	
				LOD	ρ	adjusted p-value	ρ	adjusted p-value	ρ	adjusted p-value
*Nr1h3*	2	91.18	91.20	10.85	**0.414**	**1.6E−07**	**0.532**	**1.4E−12**	**0.220**	**0.008**
*Irf7*	7	141.26	141.27	9.60	**0.389**	**1.0E−06**	**0.556**	**7.5E−14**	**0.272**	**8.7E−04**
*Batf*	12	85.69	85.71	8.68	**0.340**	**2.5E−05**	**0.560**	**4.2E−14**	**0.325**	**5.4E−05**
*Nr1h2*	7	44.55	44.55	8.51	**0.296**	**3.0E−04**	**0.456**	**3.9E−09**	**0.177**	**0.036**
*Max*	12	76.94	76.96	8.31	**0.259**	**0.002**	**0.508**	**2.4E−11**	**0.352**	**1.1E−05**
*Tcf7*	11	52.25	52.28	9.15	**0.230**	**0.006**	**0.321**	**6.9E−05**	0.111	0.204
*Tead2*	7	45.22	45.23	9.34	**0.221**	**0.008**	**0.442**	**1.3E−08**	**0.335**	**3.1E−05**
*Hmga1*	17	27.56	27.56	10.59	**0.219**	**0.009**	**0.440**	**1.7E−08**	**0.277**	**7.0E−04**
*Tcf12*	9	71.84	72.11	7.86	**0.204**	**0.015**	**0.416**	**1.2E−07**	**0.332**	**3.5E−05**
*Tgif1*	17	70.84	70.85	9.01	**0.195**	**0.021**	**0.424**	**6.0E−08**	**0.296**	**2.7E−04**
*Zfp57*	17	37.00	37.01	8.29	0.165	0.053	**0.334**	**3.2E−05**	**0.282**	**5.6E−04**
*Cxxc1*	18	74.22	74.22	8.19	0.163	0.057	**0.382**	**1.4E−06**	**0.189**	**0.024**
*Hes1*	16	30.07	30.07	8.15	0.152	0.076	**0.287**	**4.3E−04**	0.143	0.095
*Mafk*	5	139.79	139.80	7.84	0.132	0.127	**0.357**	**7.9E−06**	**0.321**	**7.1E−05**
*Klf1*	8	84.90	84.91	8.24	0.130	0.135	0.101	0.249	**− 0.170**	**0.044**
*Fosl2*	5	32.14	32.16	7.83	0.093	0.296	**0.300**	**2.2E−04**	**0.302**	**1.9E−04**
*Meis2*	2	115.86	116.07	8.74	0.082	0.358	**0.286**	**4.4E−04**	**0.195**	**0.020**
*Erg*	16	95.36	95.59	7.94	0.071	0.432	**0.257**	**0.002**	**0.217**	**0.009**
*Jun*	4	95.05	95.05	8.54	0.067	0.460	**0.242**	**0.003**	**0.307**	**1.5E−04**
*Nfyb*	10	82.75	82.76	13.38	0.055	0.544	0.106	0.223	0.120	0.168
*Mybl2*	2	163.05	163.08	18.93	0.054	0.559	0.117	0.177	0.029	0.760
*Nfyc*	4	120.76	120.83	7.90	0.044	0.631	**0.316**	**9.3E−05**	0.139	0.105
*Ehf*	2	103.26	103.30	8.13	0.034	0.720	**0.319**	**7.9E−05**	**0.279**	**6.3E−04**
*Foxk1*	5	142.40	142.46	9.47	0.028	0.767	**0.222**	**0.007**	0.047	0.608
*Maff*	15	79.35	79.36	8.17	0.026	0.788	**0.256**	**0.002**	**0.261**	**0.001**
*Tfcp2l1*	1	118.63	118.69	12.89	− 0.056	0.540	0.134	0.118	**0.190**	**0.024**
*Elk4*	1	132.01	132.03	7.94	− 0.075	0.402	0.121	0.164	0.163	0.055
*Cebpe*	14	54.71	54.71	11.71	− 0.085	0.342	0.028	0.768	0.090	0.310
*Lhx6*	2	36.08	36.11	9.40	− 0.100	0.258	0.055	0.549	0.119	0.170
*Zkscan1*	5	138.09	138.11	8.56	− 0.118	0.175	− 0.021	0.828	− 0.019	0.842
*Tfdp1*	8	13.34	13.38	10.21	− 0.125	0.152	0.034	0.715	0.158	0.063
*Pura*	18	36.28	36.29	8.13	− 0.126	0.146	0.082	0.354	**0.195**	**0.020**
*Nr2c2*	6	92.09	92.17	8.29	− 0.128	0.141	0.105	0.228	**0.173**	**0.040**
*Nr2c1*	10	94.15	94.20	8.68	**− 0.177**	**0.037**	0.124	0.153	**0.225**	**0.007**

Significant values are in bold.

Lastly, we examined direct or indirect target genes of *Nr1h3* to determine if the expression, genetic regulation, and correlation with aortic lesion area of *Nr1h3* could affect the relationship between *Nr1h3*’s target genes and atherosclerosis. Of the *Nr1h3-*dependent genes reported^27,28^, ten genes showed strong correlations with *Nr1h3* expression and atherosclerosis or plasma lipids in our DO-F1 cohort (Fig. 6D). These genes are involved in cholesterol metabolism (*Abca1*, *Abcg8*, *Abcg5*, *Lpl*, *Cyp7a1*, and *Pltp*), lipid and atherosclerosis (*Abca1*, *Il1b*, *Ccl2*, and *Abcg1*), bile secretion (*Abcg8*, *Abcg5*, and *Cyp7a1*), and IL-17 signaling pathway (*Ccl7*, *Il1b*, and *Ccl2*) (Table S12). These results demonstrate that genetic variation in DO mice helped identify Nr1h3 which has mechanistic relationships with dyslipidemia and atherosclerosis. Furthermore, this implies that genetic variation of this, and potentially additional, TF contributes to the complexity of atherosclerosis.

## Discussion

In this study, we observed a significant effect of genetic factors on atherosclerosis and investigated how liver gene expression and genetic regulations interact to affect these traits. Several conclusions were drawn from this study. First, we identified predominant sexual dimorphism and tremendous phenotypic variation in the atherosclerotic traits. Second, QTL analysis of hyperlipidemic DO-F1 mice identified genetic regulation of atherosclerosis and plasma lipid levels. These include identification of an aortic lesion area QTL narrowing the boundaries of a previously identified loci on Chr 10^29^ and a novel QTL on Chr 19 perhaps reflecting sex-specific genetic regulation^29^. Third, integration of gene expression, genetic regulation, and gene-trait correlation discovered multiple hepatic candidate genes and liver TFs that associated with atherosclerosis in DO-F1 mice. These results predict a prominent role of putative genes in the modulation of atherosclerotic traits. Each of these points is discussed in turn below.

Identification of sex-specific QTLs for aortic lesion area demonstrates the complexity of atherosclerosis in DO-F1 mice. Sex differences in aortic lesion area and sex-specific QTLs have previously been reported in F2 cross studies^30,31^. Our results reveal that aortic lesion area QTLs which were identified in DO-F1 mice show sex bias. These include a higher resolution of aortic lesion area QTL (confidence interval: ~ 8 Mbp) than the previous study (confidence interval: ~ 51 Mbp) that identified loci on Chr 10^29^ in females and a novel QTL on Chr 19 in males. The sex specificity of these QTLs reiterates that the genetic regulation of atherosclerosis is complex, and that the expression of candidate genes likely differs between sexes. This has important implications for development of therapies for atherosclerosis in men and women as the underlying genetic effects and the genes they affect may differ between sexes. This study supports the notation that a detailed investigation of sex differences and their underlying mechanisms are critically needed as we move towards “personalized medicine.”

Genetic regulation of the liver transcriptome also contribute to complex traits, and colocalization analysis between *cis*-eQTLs and atherosclerosis revealed genetic variant-gene-atherosclerosis associations. One of the main goals of our study was to discover novel genes that influence atherosclerosis, and we hypothesized that these genes would reveal new pathways and mechanisms. To identify robust candidate genes associated with atherosclerosis, we considered allele effects, *cis*-eQTL, and correlation with lesion size. Since eight alleles are segregated in DO mice, high-resolution mapping allowed us to investigate whether each allele was associated with lesion size. Several notable genes were identified as positional candidate genes based on the genetic regulation of their expression (*cis*-eQTL) and also correlated with aortic lesion area. Two candidate genes, *Ptprk* and *Pten*, encode protein tyrosine phosphatases (PTPs). PTPs are important modulators of cellular processes such as migration, proliferation, as well as differentiation and are involved in pathological changes of vascular wall function^32^. For example, *Ptprk* has previously been shown to associate with colorectal cancer^33^, angiogenesis^34^, and cell–cell contacts in epithelial cells^35^. Additionally, *Ptprk* possesses other unclear biological functions. For example, human genome-wide association studies have shown that *Ptprk* SNPs are associated with athletic performance and risk of Celiac disease^36,37^. Furthermore, data from approximately 500,000 individuals demonstrate that *Ptprk* is associated with LDL-C, platelet function tests, and inflammatory bowel disease (NCBI’s Phenotype-Genotype Integrator https://www.ncbi.nlm.nih.gov/gap/phegeni). *Pten*, another candidate with overlapping *cis*-eQTL and atherosclerosis QTL, is a tumor suppressor gene that is expressed in endothelial cells, sub-endothelial cells, and vascular smooth muscle cells^38^. *Pten* has previously been shown to influence the development of atherosclerosis^38,39^. In mice, *Pten* overexpression reduces plaque area and preserves contractile protein expression in smooth muscle cells (SMC) in atherosclerosis^38^. *Pten* deletion promotes spontaneous vascular remodeling in SMC and *Pten* reduction correlates with increased atherosclerotic lesion severity in human coronary arteries^39^. This relationship between *Pten* expression and atherosclerosis is consistent with the correlation analysis in the DO-F1 cross. In our analysis, *Pten* gene and lesion size showed a significant negative correlation only in males, which is also consistent with previous reports, considering that most functional validations were performed in male mice^39^. Lastly, *Cyp2c67* is another candidate with overlapping *cis*-eQTL physically located within the Chr 19 atherosclerosis QTL, but whose function itself has not been extensively characterized. However, cytochrome P450s (CYPs), which include *Cyp2c67*, catalyze the metabolism of drugs and substrates in liver and have been shown to be associated with the pathogenesis of atherosclerosis in many studies. In human GWAS studies, polymorphisms of CYPs have been reported to confer disease protection or reduce risk of CVD and atherosclerosis^40^. In animal models, CYPs were markedly inhibited in atherosclerosis-induced rabbits and mice lacking Niemann-Pick type C, which alleviates atherosclerosis by regulating macrophage intracellular cholesterol trafficking^41,42^. This supports that CYPs may provide a protective effect on atherosclerotic lesion development. Taken together, these findings and reports provide strong evidence that the candidate genes found in this study may have a pivotal role in the liver towards atherosclerosis development in mice and humans.

Feeding the HFHC diet feeding induces the expression of multiple genes, including specific transcription factors (TFs) which activate specific proinflammatory pathways^43^. In the current study, the integration of liver TF binding sites with gene expression and genetic regulation yielded associations between atherosclerosis and specific TFs. Of the 265 TFs expressed in DO-F1 liver tissue, eleven genetically regulated TFs were correlated with atherosclerosis, and our results not only suggest that lipid metabolism and inflammatory pathway-related TFs regulate gene expression For example, the liver X receptor alpha (LXRα), encoded by *Nr1h3* gene, is an important regulator of cholesterol, fatty acid, and glucose homeostasis^44^. LXRα expression is highest in the liver among all organs^45^, and LXRα target genes, ATP binding cassette (ABC) transporters A1/G1, apolipoprotein E/CI, and members of the Cyp7a family are also highly expressed in the liver of both humans and mice^27,44^. Numerous studies have shown that LXRα is involved in many pathways underlying the development of atherosclerosis and CVD, including lipid metabolism, innate immunity, and inflammation^45,46^. Our study showed that, among the target genes of LXRα, key lipid processing genes including *Abcg1*, *Lpl*, *Pltp*, and *Abca1* have a strong positive correlation with atherosclerosis. Furthermore, using the Harmonizome database (https://maayanlab.cloud/Harmonizome^47^), we found that there are putative 597 target genes that bind to the *Nr1h3* transcription factor using the CHEA Transcription Factor Targets dataset, and *Pten* is the only target gene of *Nr1h3* among the 86 liver expressed genes discovered in the aortic lesion area QTL in our DO-F1 cohort. While the relationship between *Pten* and *Nr1h3* we observed in the liver expression has not been reported to date, both *Pten* and *Nr1h3* are expressed widely including in vascular cells and macrophages. Further studies are fruitful to determine how the interaction between *Pten* and *Nr1h3* affects the pathogenesis of atherosclerosis beyond plasma atherogenic traits. Taken together, these systems genetics-based integration analyses helped us elucidate the origin of the gene-atherosclerosis association, as hypothesized for the effects of candidate TFs on atherosclerosis. Associating genetic variants with the expression of TFs and their target genes can identify novel mechanistic basis of disease risk and improve our understanding in precision medicine for atherosclerosis^48,49^. Understanding the influence of environmental factors and diet on genetic risk is critical to developing individualized therapies and recommendations.

The study also showed several limitations of our approach, similar to other QTL approaches. Despite the reduced number of test subjects required by using DO mice compared to human GWAS studies, DO mouse studies may still require large numbers of mice when targeting complex traits, including atherosclerosis. For example, we did not obtain any significant QTLs that were associated with atherosclerosis in the sex additive model or plasma total cholesterol in the three models. The effect size of phenotypic variation is an important consideration when determining sample size. While the risk of complex diseases of individuals is determined by the combination of small changes in multiple genes, genetic variation of the expression levels of a given gene in our DO-F1 cohort range at best 50% to 200% normal, even assuming heterozygous for a null allele or for a threefold over expressing allele. Consequence of allelic protein structures in heterozygous state would be also small unless one allelic variant protein pairing with the other affects dominantly or dominant-negatively. Although knowledge of physical interaction of a protein with other proteins forming large complexes has increased dramatically, finding effects of a single gene change in complex traits is still difficult. In this regard, our detection of the significant QTLs on chr10 and chr19 might have been facilitated by the clustering of multiple candidate genes correlated with atherosclerotic traits significantly to the same direction. Corollary to this is that our approach has likely missed other atherogenic genes simply because of their individually small effects or because multiple protective and promotive genes are clustered in small regions. Our data set would provide an excellent resource to explore these and other concepts in future studies.

A comment is required on the atherosclerosis sensitizer models for study. Our DO-F1 models were sensitized by carrying human *CETP* and *ApoE3-Leiden* transgenes, and we observed the liver expression of the retinol-binding protein 4 (*Rbp4*)) gene within the Chr19 QTL exhibiting a strong negative correlation with plaque size. This suggests that RBP4 has protective effects. However, Liu et al.^50^ recently reported that adenovirus-mediated overproduction of *Rbp4* increases atherosclerosis burden in apoE-deficient mice. These two models exhibit similar atherogenic lipoprotein patterns on high fat/high cholesterol diet, but underlying mechanisms differ. Additionally, expression variations in DO-F1 is likely within physiological ranges, while adenovirus-mediated effects are large in both directions. Future studies of *Rbp4* are necessary.

Complex traits can also be determined by the interaction of multiple genes. Although human GWAS studies and DO mouse QTL studies are likely to reveal multiple loci contributing to the observed phenotype, functional validation of candidate loci or genes is still required. Two of our putative candidate genes identified in this study (i.e. *Pten* and *Nr1h3*) have already been validated for atherosclerosis^39,46,51^. Our colocalization approach using liver tissue-derived expression limits candidate genes primarily to lipid metabolism. Future studies are necessary to examine candidate genes through transcriptome analysis such as in aortic tissue where atherosclerosis develops. Lastly, utilization of the DO-F1 cross rather than DO mice reduces the degree of freedom of genotype prediction from 36 to 8 due to the use of only 1 chromosome for genetic mapping. This affects the complexity of genetics but also can affect the strength of associations for additive alleles and may also eliminate the ability to detect recessive associations^52^. In this regard, QTL hits on the chrX may be sex-biased since one sex can only have a DO chrX while the other sex has both a copy of the DO chrX and a copy from their C57BL/6J sires or dams.

To date, the use of the DO-F1 cross has been reported for prostate cancer and tumor immunity^52,53^, and our study is the first study using the DO-F1 cross for atherosclerosis. We previously identified the aortic lesion area QTL (CI: 0.2 Mbp) with high resolution in a model fed high fat and cholic acid diet in DO female mice, and identified *Apobec1* gene as a candidate gene that associated with atherosclerosis^54^. However, due to the physiological characteristics of mice, where atherosclerosis is not well developed by diet alone, we expected to induce atherosclerosis by making F1 generation and to discover aortic lesion area QTL(s). Our QTL results reflect the high sexual dimorphism and the complexity of atherosclerosis itself.

## Conclusion

In this study, we used F1 crosses of DO mice to deepen our understanding of atherosclerosis. This is the first comprehensive study of the integrated analysis of the atherosclerotic traits and liver transcriptome in DO-F1 mice. We provide evidence for genes associated with atherosclerosis, such as revealing an association with atherosclerosis for *Ptprk*, *Pten*, *Cyp2c67,* and *Nr1h3*. Specifically, integration of multiple data led to a mechanistic view of how a variation in the *Nr1h3* TF could affect atherosclerosis. A limitation of this study is that liver tissue, not aorta, was used to discover candidate gene expressions associated with atherosclerosis. Further studies at the genetic, regulatory, and functional levels of liver genes discovered herein will enhance our understanding of liver physiology and its role in disease states, as well as provide insight for potential therapeutic means.

## Methods

### Ethics statement

We followed all NIH animal welfare guidelines and animal care and study protocols were approved by the University of California Davis’ Institutional Animal Care and Use Committee (IACUC). All experiments were performed in accordance with the relevant guidelines and regulations within ARRIVE (Animal Research: Reporting of In Vivo Experiments) guidelines.

### Animals: hyperlipidemic DO-F1 mice

Animal care and study protocols were approved by the University of California Davis Animal Care and Use Committee. 6-week-old CETP/ApoE3 Leiden males, hemizygous to the CETP and ApoE3 Leiden transgenes (Tg), were kindly provided by Dr. Aldons Lusis^14^. In addition, a total of 200 F0 J:DO females (JAX stock number 009376, outbreeding generation # 26,28) were crossed with CETP/ApoE3 Leiden males to produce 238 female and 234 male (J:DO x CETP/ApoE3 Leiden) F1 offspring. DO-F1 progeny were genotyped by PCR to confirm the presence of CETP and ApoE3-Leiden transgenes^14^ and randomly selected from litters. After weaning, all selected DO-F1 mice were maintained on a synthetic diet, AIN-76A (D10001, Research Diets, New Brunswick, NJ). After the age of 8 weeks, all mice were fed with a synthetic high-fat and high-cholesterol (HFHC, 33% kcal from fat, primarily cocoa butter, and 1.25% cholesterol) diet (Research Diets D121083) (Table S1) ad libitum. After 16 weeks on HFHC diet, mice were fasted for 4 h and blood was collected. Mice were then euthanized by cervical dislocation after anesthesia with 1.5% isoflurane and tissues collected (reference). Animals were maintained on a 12 h light and dark cycle under temperature- and humidity-controlled conditions. Euthanasia of all mice was performed by cervical dislocation after anesthesia with 1.5% isoflurane and their body temperature was kept at 37 °C. Following a 4 h fast, blood was collected from the retro-orbital plexus into plasma collection tubes with EDTA (Becton Dickinson, Franklin Lakes, NJ). Blood was kept on ice, and the separated plasmas by centrifugation were frozen at − 80 °C in aliquots prior to analysis.

### Plasma clinical atherosclerotic traits

Plasma levels of TC, HDL-C, TG, and glucose were quantitated at 24 weeks of age using a COBAS INTEGRA 400 plus Analyzer (Roche Diagnostics, Indianapolis, IN, USA). Fifty microliters (50 μL) of plasma sample were diluted three times with 1 × phosphate-buffered saline (PBS) and processed using standard procedures according to the analyzer's instructions. HDL-C was subtracted from TC to determine VLDL-C/LDL-C levels.

### Atherosclerotic lesion size

The lesion area of the proximal aorta was performed as described in previous studies^14,55^. Hearts containing the proximal aorta were dissected in 24-week old mice, perfused with 1 × PBS, and stored in 10% formalin at 4 °C. The superior portion of the heart, including the aortic sinus and a portion of the atria, were isolated by a transverse cut parallel to the atria, and then embedded in the optimal cutting temperature compound and stored at − 80 °C. Consecutive sections (10 μm thick) from the aortic sinus were mounted on slides by the UNC Histology Research Core Facility, and lesion area was quantified from every eighth section to the proximal aorta. A total of 80 sections were collected from each mouse. Aortic lesion areas were quantified using Aperio’s ImageScope (Vista, CA). Data were presented as the mean lesion area in μm^2^.

### Mouse genotyping and haplotype reconstruction

Genotyping was performed in tail biopsies using the Mouse Universal Genotyping Array (GigaMUGA, 143,259 markers) by Neogen (Lincoln, NE)^56^. Identified genotypes were converted to founder strain–haplotype reconstructions using R/QTL2 software^57^. GigaMUGA markers were interpolated to an evenly spaced grid at 0.02-cM intervals, and markers were added to fill the areas that physically represent the sparse. A total of 461 genotypes (235 females and 226 males) were used for mapping and haplotype reconstruction after verifying the probe intensities for SNPs on the sex chromosomes, calculating the proportion of matching SNP genotypes for each pair of samples and eliminating > 10% missing genotypes via genotype data cleaning^58^. By constructing allele probabilities for DO-F1, we confirmed that allele frequencies of the 8 founder alleles were almost identical across the genome (Fig. S5).

### RNA-Seq sample preparation and quantification

Samples were selected based on the aortic lesion area levels; we focused on mice with high aortic lesion size and half of the mice with low aortic lesion size were selected from each sex. Among 77 males and 85 females, there were 38 males and 42 females with high lesion size and 39 males and 43 females with low lesion size. Details of RNA processing are described in the supplementary information. High quality RNA samples from 85 females and 77 males were submitted to the UC Davis DNA Technologies Core. To minimize technical variability, all samples were assigned to each lane and the pooled libraries were sequenced on two lanes of the Illumina NovaSeq 6000 sequencing platform (Illumina Inc., San Diego, CA, USA) to achieve at least 25 million paired-end reads of 150 bp. Raw data were deposited at National Center for Biotechnology Information’s Gene Expression Omnibus (GEO accession GSE179091). Details of RNA alignment are described in detail in the supplementary information. After alignment, we filtered in 13,094 genes (381 X-linked, 7 Y-linked, 15 mitochondrial genes, and 12,691 autosomal genes) with mean transcripts per million (TPM) greater than 1 in 162 liver samples. This TPM filter was used to remove genes that were only expressed at low levels. We normalized the filtered genes by the upper quartile value to account for differences in library size and transformed them to rank normal scores using the 'rankZ' function in the DOQTL R package^57^ for the eQTL analysis.

### Quantitative trait loci mapping for aortic lesion area and transcripts

QTL mapping was analyzed using the R (v3.5.3) package R/QTL2 (v0.20)^59^. Genome scans for atherosclerotic traits and transcripts were performed in three different models using the “scan1” function in R/qtl2: (1) sex additive—sex and generation number were included as additive covariates, (2) female mice—generation number was included as an additive covariate, and (3) male mice—generation number was included as an additive covariate. SNPs in the candidate genes found in QTL were identified based on the Wellcome Trust Sanger mouse genomes database (www.sanger.ac.uk), release 1410, based on genome assembly GRCm38^60^.

In this study, significant atherosclerotic trait QTL and eQTL thresholds were determined using the scan1perms function^59^ in qtl2 with 1,000 permutations. For atherosclerotic trait mapping, traits with a Shapiro–Wilk W value ≥ 0.95 were considered as normalized data. Non-normal phenotypic traits were log2 transformed and the aortic lesion area was transformed to rank normal scores using the 'rankZ' function in the DOQTL R package. eQTL was defined as *cis*-eQTL when the SNP with the maximum LOD score was within ± 4 Mb at the transcription start site, and trans-eQTL was defined when this condition was not met. The genetic architecture of chrX in the DO-F1 mouse model is complex and our breeding scheme allows for males to have only a DO chrX while females have both a copy of the DO chrX and a paternal copy from their C57BL/6 J sires, we focused on the autosomal QTLs consistent with previously reported DO-F1 study^53^.

### Heritability

The extent to which phenotypic variation is affected by genotypic variation, a linear mixed-effect model was used to estimate the narrow-sense heritability scores of the atherosclerotic traits and liver transcriptome. This was performed using the function “est_herit” in R/qtl2 by submitting a kinship matrix and each trait value.

### Identification of liver transcription factors associated with CVD-related GWAS

First, we prioritized 453 mouse TFs that were identified by large-scale chromatin immunoprecipitation sequencing analysis in promoter regions using the HOCOMOCO v11^26^ and primarily filtered 265 TFs expressed in liver in DO-F1 mice. We used Phenotype–Genotype Integrator (http://www.ncbi.nlm.nih.gov/gap/phegeni)^61^ to prioritize liver TFs to generate biological hypotheses based on published CVD GWAS and meta-analyses studies. By searching this public database with the 60 CVD-related phenotypes such as “Coronary Artery Disease”, “Myocardial Infarction”, “Stroke”, “Cholesterol”, “Triglyceride” and “Metabolic Syndrome X”, we found relevant CVD-associated SNPs, genes, and association results. The association with P < 1 × 10^–7^ was chosen to select as candidate SNPs.

### Other statistical analysis

All statistical analyses were performed in R (v.3.5.3) (R Core Team). Spearman’s correlation was used to correlate the atherosclerotic traits and liver transcripts (log2TPM). The p-values were adjusted using the BH false discovery rate (FDR) procedure^62^, and correlation coefficients and adjusted p-values were visualized using the ‘pheatmap’ package^63^. Significance was determined with a p-value < 0.05. Summary statistics were calculated to evaluate the magnitude of variability of the atherosclerotic traits.

## Disclaimer

The USDA is an equal-opportunity employer.

## Supplementary Information

Supplementary Figures.

Supplementary Information 1.

Supplementary Tables.

## Data Availability

RNA sequencing data for 162 samples within this study were deposited at National Center for Biotechnology Information’s Gene Expression Omnibus (GEO accession GSE179091).
