# Supplementary material for: Integrative analysis of hepatic transcriptional profiles reveals genetic regulation of atherosclerosis in hyperlipidemic Diversity Outbred-F1 mice
Source: Sci Rep. 2023 Jun 10;13:9475. doi: 10.1038/s41598-023-35917-8 (PMC10257719; doi:10.1038/s41598-023-35917-8)
Supplement: Supplementary file 1 — Supplementary Figures. [file 41598_2023_35917_MOESM1_ESM.pptx]

## Slide 1
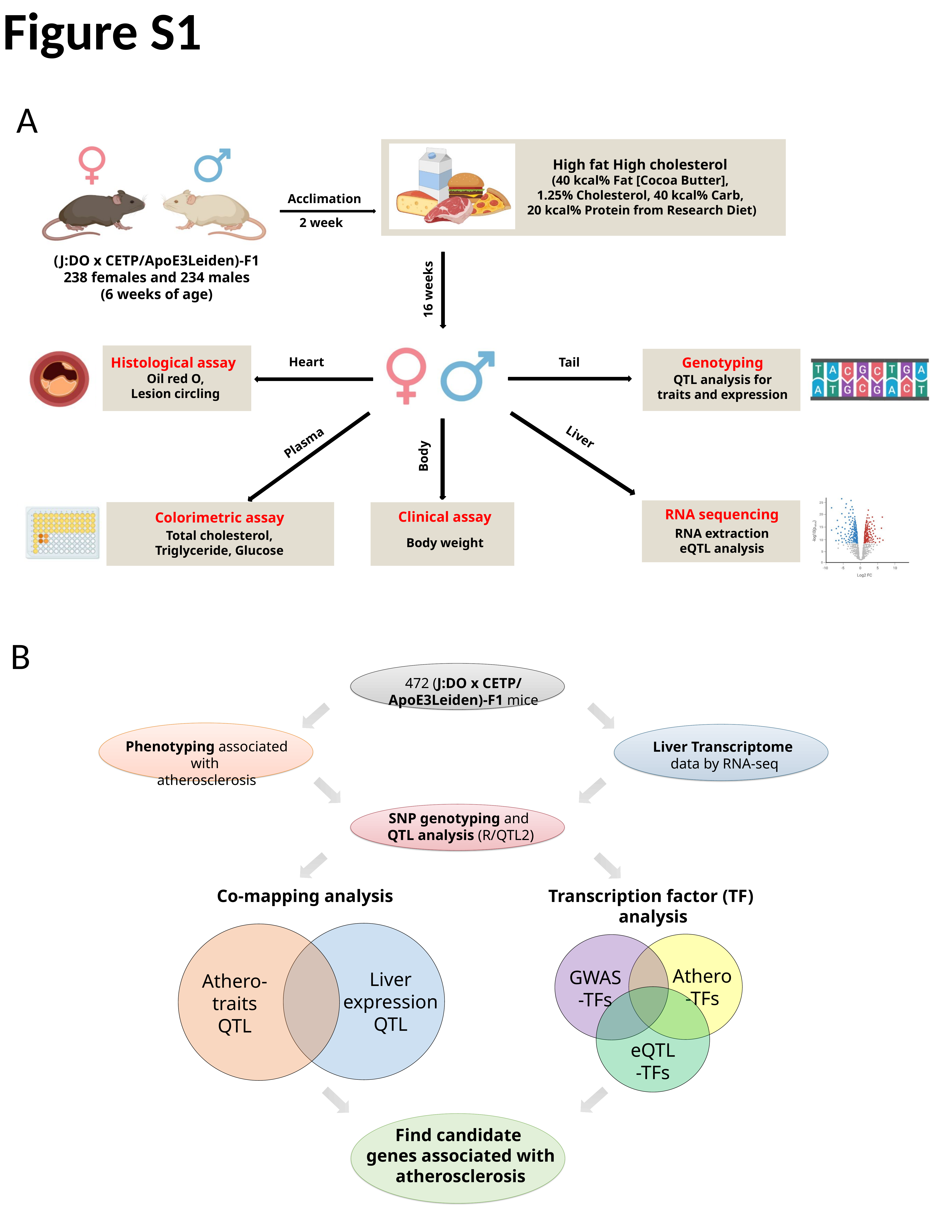

Figure S1
A
High fat High cholesterol
(40 kcal% Fat [Cocoa Butter],
1.25% Cholesterol, 40 kcal% Carb,
20 kcal% Protein from Research Diet)
Acclimation
2 week
(J:DO x CETP/ApoE3Leiden)-F1 238 females and 234 males
(6 weeks of age)
16 weeks
Genotyping
QTL analysis for
traits and expression
Histological assay
Heart
Tail
Oil red O,
Lesion circling
Liver
Plasma
Body
RNA sequencing
RNA extraction
eQTL analysis
Clinical assay
Colorimetric assay
Total cholesterol, Triglyceride, Glucose
Body weight
B
472 (J:DO x CETP/ApoE3Leiden)-F1 mice
Phenotyping associated with
atherosclerosis
Liver Transcriptome
data by RNA-seq
SNP genotyping and
QTL analysis (R/QTL2)
Co-mapping analysis
Transcription factor (TF) analysis
Liver
expression
QTL
Athero-
traits
QTL
Athero
-TFs
GWAS
-TFs
eQTL
-TFs
Find candidate
genes associated with atherosclerosis

## Slide 2
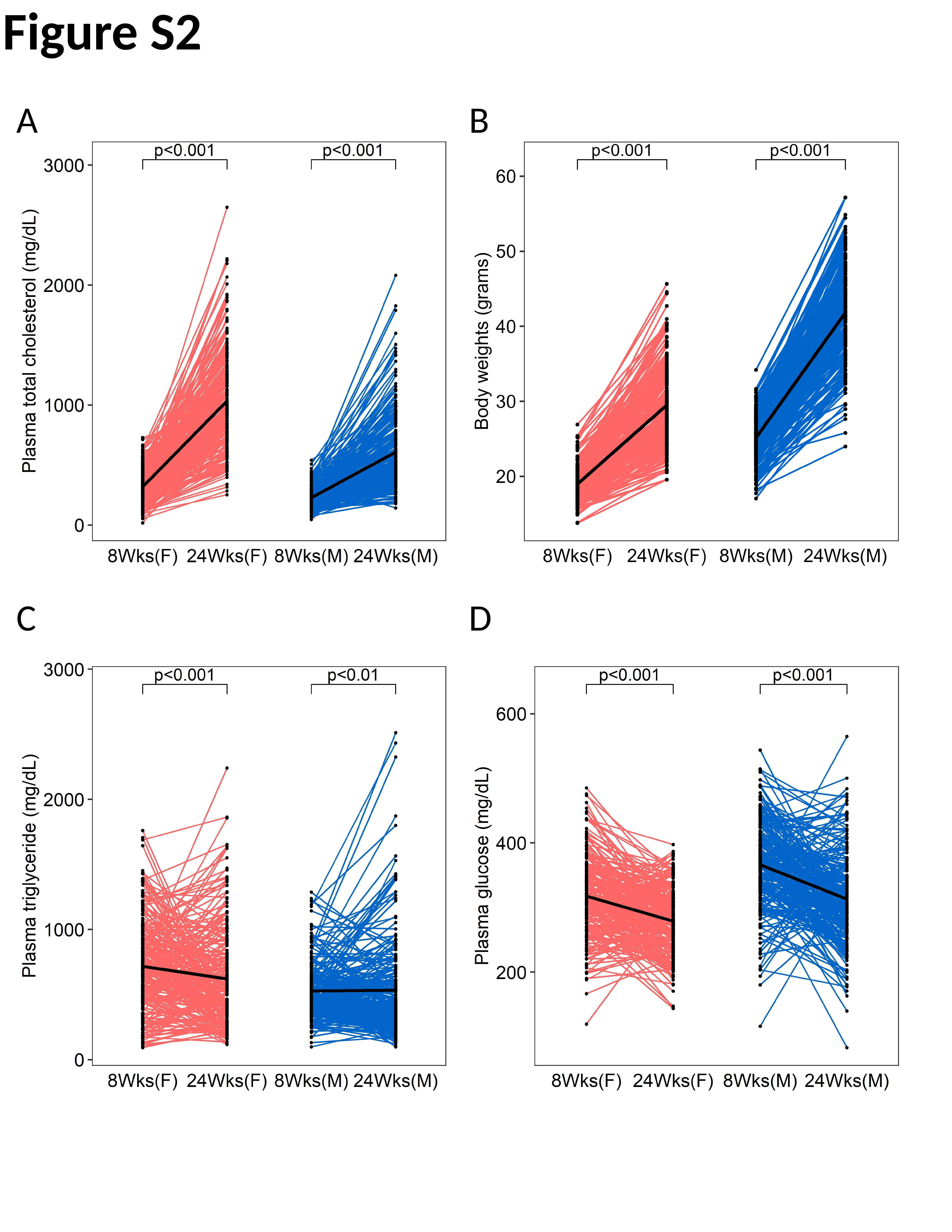

Figure S2
A
B
C
D

## Slide 3
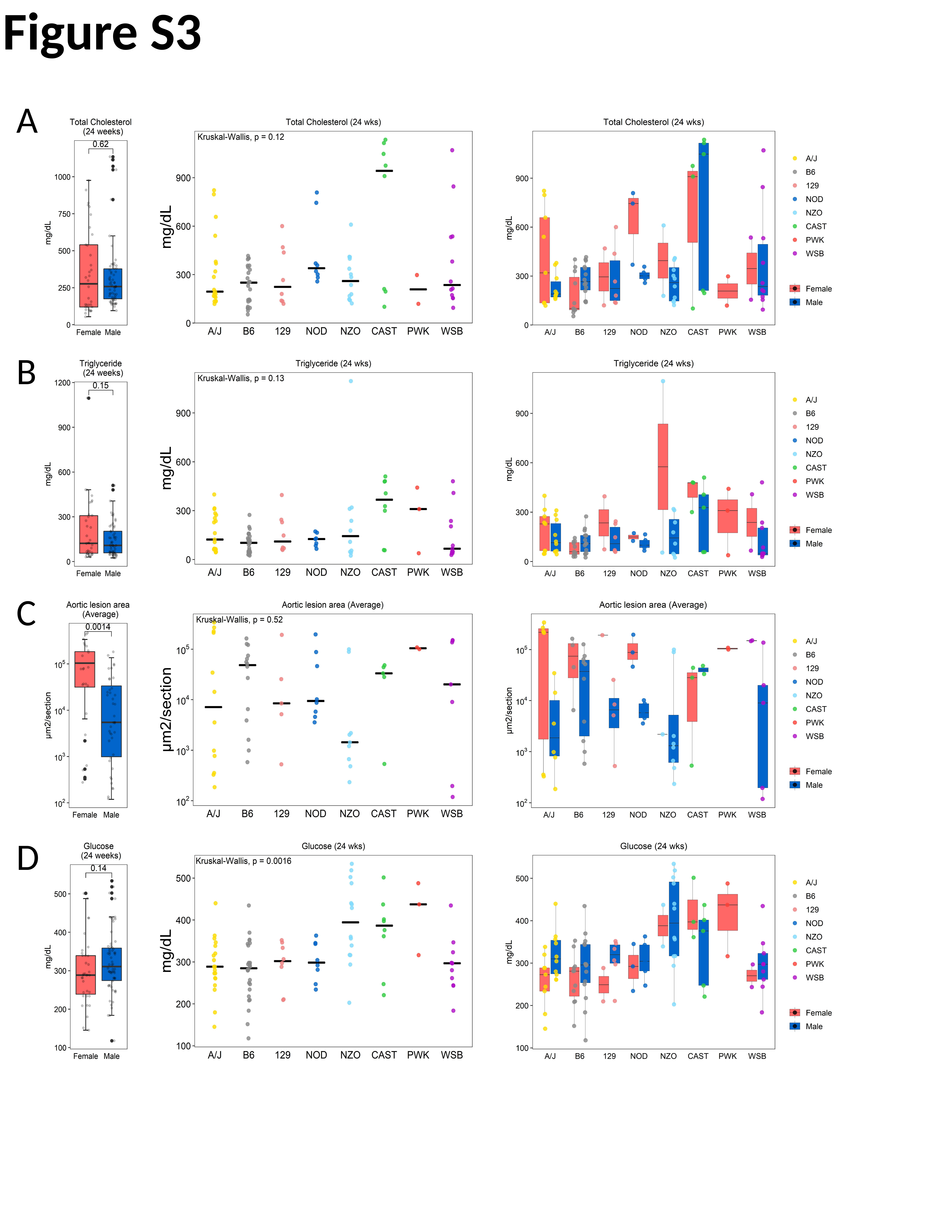

Figure S3
A
B
C
D

## Slide 4
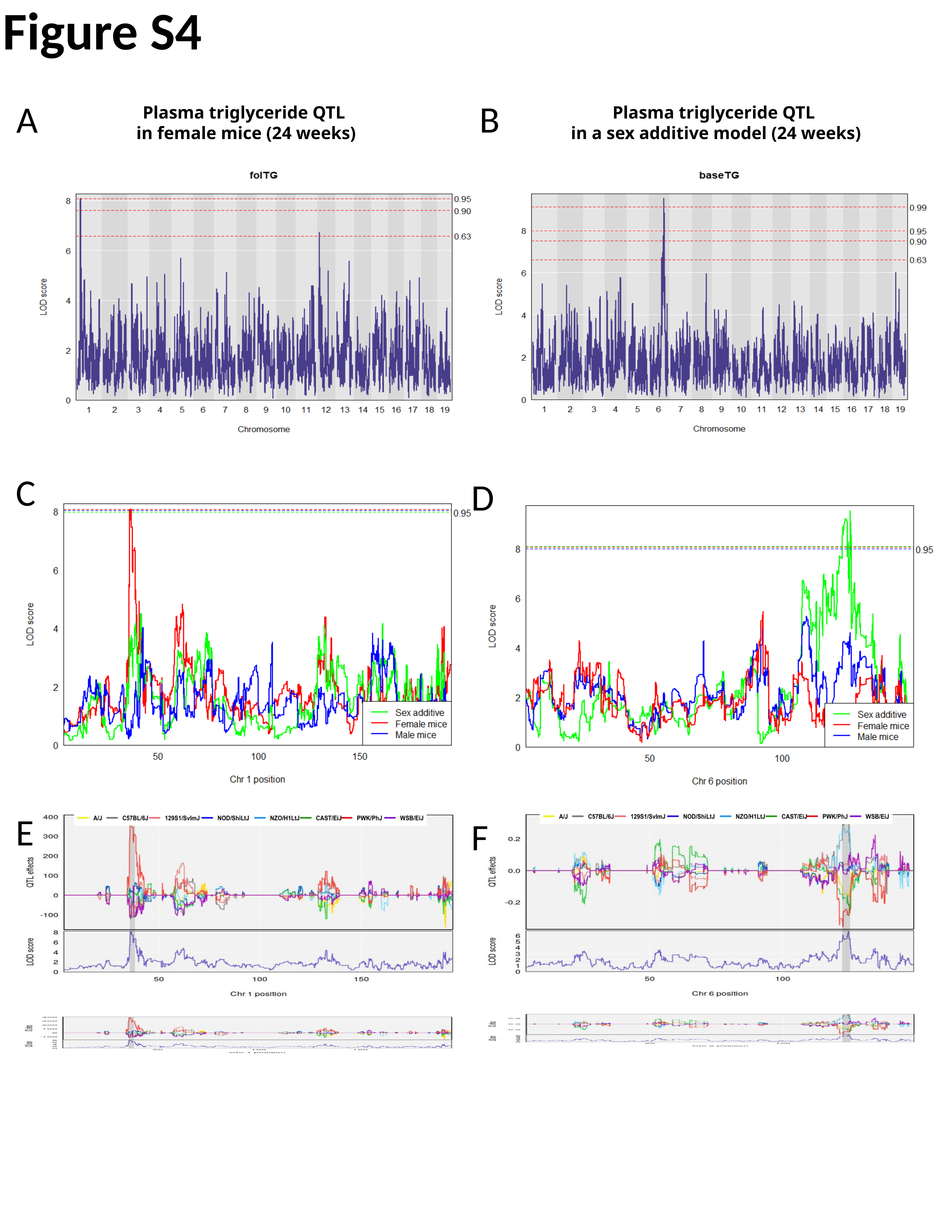

Figure S4
B
A
Plasma triglyceride QTL
in female mice (24 weeks)
Plasma triglyceride QTL
in a sex additive model (24 weeks)
C
D
E
F

## Slide 5
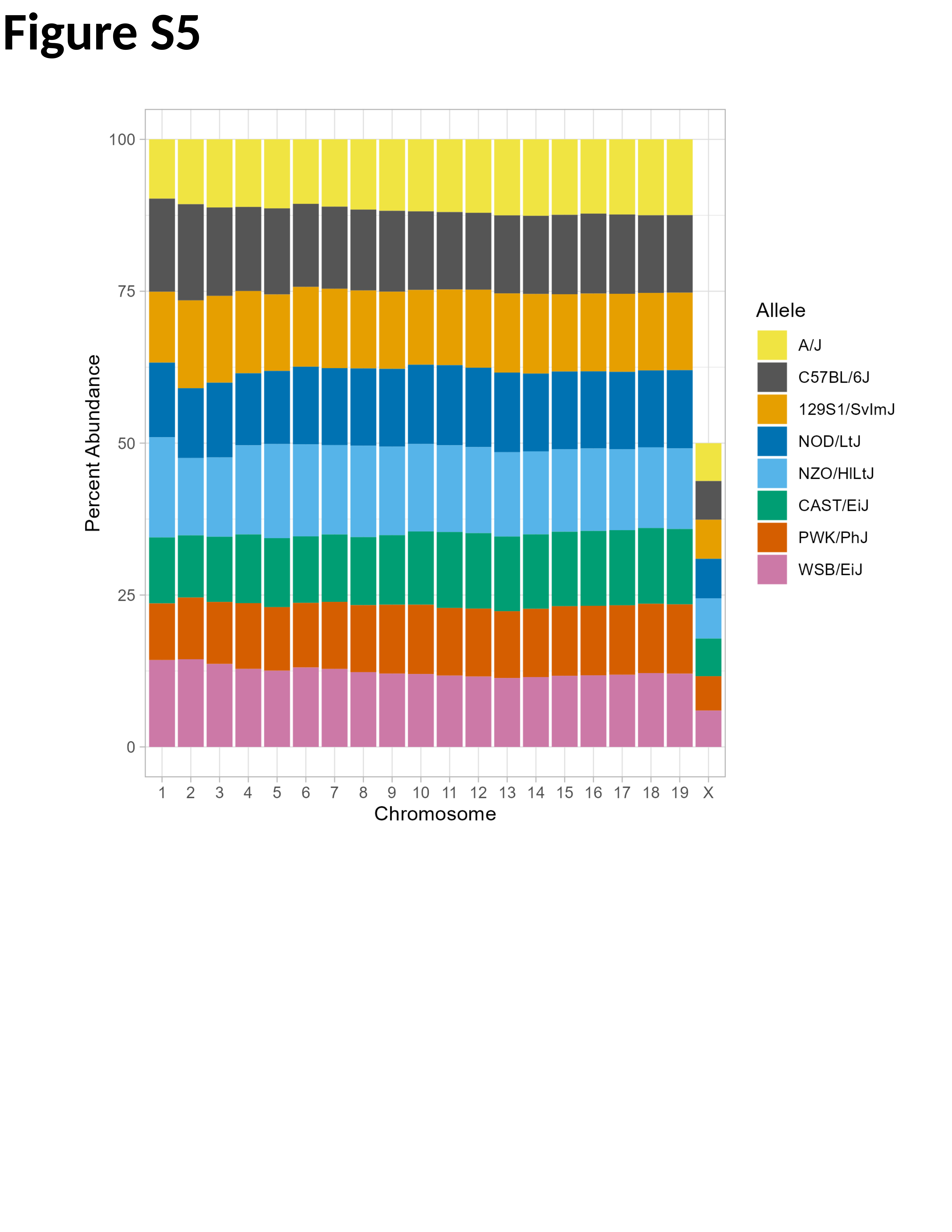

Figure S5
