# Supplementary material for: Integrative analysis of hepatic transcriptional profiles reveals genetic regulation of atherosclerosis in hyperlipidemic Diversity Outbred-F1 mice
Source: Sci Rep. 2023 Jun 10;13:9475. doi: 10.1038/s41598-023-35917-8 (PMC10257719; doi:10.1038/s41598-023-35917-8)
Supplement: Supplementary file 2 — Supplementary Information 1. [file 41598_2023_35917_MOESM2_ESM.docx]

**Supplementary Information**

**Integrative analysis of hepatic transcriptional profiles reveals genetic regulation of atherosclerosis in hyperlipidemic Diversity Outbred-F1 mice**

Myungsuk Kim^1,4,5^, M. Nazmul Huda^1,2^, Levi Evans^2^, Excel Que^2^, Erik R. Gertz^2^, Nobuyo Maeda-Smithies^3^, Brian J. Bennett^1,2^

^1^Department of Nutrition, University of California, Davis, CA

^2^ Western Human Nutrition Research Center, Agricultural Research Service, US Department of Agriculture, Davis, CA

^3^Department of Pathology and Laboratory Medicine, University of North Carolina at Chapel Hill, Chapel Hill, NC

^4^Korea Institute of Science and Technology (KIST), Gangneung, Gangwon-do, Republic of Korea

^5^ Division of Bio-Medical Science and Technology, KIST School, University of Science and Technology (UST), Seoul, 02792, Republic of Korea.

**Supplementary methods:**

### Animals: Hyperlipidemic Eight DO Founder Strains-F1 Mice

Animal care and study protocols were approved by the University of California Davis Animal Care and Use Committee. For the strain survey experiment, we crossed CETP/ApoE3 Leiden males to one of eight strains to generate eight different F1 strains of mice (AJ-F1, B6-F1, 129-F1, NOD-F1, NZO-F1, CAST-F1, PWK-F1, and WSB-F1, respectively) and quantified atherosclerotic traits in F1 female and male mice. Three females from each of the eight DO founder inbred strains (five classical laboratory inbred strains [A/J, B6, 129, NOD and NZO] and three wild-derived inbred strains [CAST, PWK, and WSB]) and DO females were obtained from The Jackson Laboratory (Bar Harbor, ME).

### RNA Library Preparation and Sequencing

Total RNA was extracted from snap-frozen liver using miRVana total RNA isolation kit (Thermo Fisher Scientific, Waltham, MA, USA) according to the manufacturer’s protocol. The quality and amount of liver RNA were evaluated using a Bioanalyzer (Agilent, Inc., Santa Clara, CA). The average RNA-integrity score for 162 DO-F1 liver samples was 9.01 ± 0.4. The RNA-seq libraries were constructed from 1 µg total RNA after poly-A library preparation.

### RNA-Seq Mapping and Quantification

Raw read data were filtered using HTStream (version 1.1.0, <https://github.com/ibest/HTStream>), which included screening for contaminants (such as PhiX and rRNA), PCR deduplication readout, quality-based trimming, adapter trimming, sex-specific technical biases and overlapping paired-end reads. Custom R code was then used for sequence read and alignment quality assessment (**Table S8**). Genetic variants such as SNPs and insertions/deletions (Sanger REL-1410) in eight founder strains were incorporated into the B6 reference strain genome (GRcm38/mm10) to generate strain-specific genomes. After obtaining transcript sequences of all annotated genes in each 8-founder strain genome, the allele sequences for each transcript were incorporated into one pooled transcriptome for read alignment. After alignment, the expected read counts obtained from each transcript allele were quantified using an expectation maximization algorithm (EMASE, <https://github.com/churchill-lab/emase>) ^1^.

### Quantitative Trait Loci Mapping for Aortic Lesion Area and Transcripts

The genome scan models incorporated random effects to adjust for genetic relatedness between DO-F1 mice using the LOCO method ^2^. Reported mapping statistics were LODs, and confidence intervals for QTL were calculated as 95% Bayesian credible intervals ^3^. Using a linear mixed model with allele probabilities as random effects, the association between the trait and each founder strain genotype in each QTL was determined using Best Linear Unbiased Predictor. The significance threshold at P < 0.05, the highly suggestive threshold at P < 0.1 and the suggestive threshold at P < 0.63 of all reported QTLs were empirically determined by permutation analysis ^4,5^.

**Supplementary Figure legends**

**Figure S1. Study design and timeline for Diversity Outbred (DO)-F1 mice.**

A total of 200 F0 J:DO females (JAX stock number 009376, outbreeding generation # 26,28) were crossed with CETP/ApoE3 Leiden males to breed 238 (CETP/ApoE3 Leiden × J:DO) F1 females and 234 (CETP/ApoE3 Leiden × J:DO) F1 males. Female and male progeny were genotyped to confirm the presence of CETP and ApoE3-Leiden transgenes and maintained on a synthetic diet, AIN-76A until 8 weeks of age. At the age of about 8 weeks, all mice were fed with a synthetic high-fat and high-cholesterol (33 kcal % fat from cocoa butter and 1.25% cholesterol) diet ad libitum. Mice were euthanized for tissue collection after fed this diet for 16 weeks.

**Figure S2. Spaghetti plots of plasma lipids, glucose and body weight at 8 and 24 weeks in each sex.** Plasma total cholesterol (A) and body weight (B) increased in most mice after 16 weeks of HFHC diet challenge. Plasma triglyceride (C) and glucose (D) were slightly decreased on average at 24 weeks of age but displayed a variable response for individual mice. The p-values calculated from Wilcoxon signed-rank test between time points within each sex.

**Figure S3. Effects of sex and genetic backgrounds in atherosclerotic traits in DO founder strains-F1 mice at 24 weeks**. Eight DO founder strain females were crossed with CETP / ApoE3 Leiden males to breed 105 (CETP/ApoE3 Leiden × eight DO founder strains) F1 mice for the strain survey experiments. At the age of about 8 weeks, all mice were fed with a synthetic high-fat and high-cholesterol (HFHC) diet for 16 weeks. Aortic lesion area is higher in females in eight DO founder strains-F1 mice. The p-values were Wilcoxon signed-rank test for aortic lesion area. n = 102 (by sex- #females= 37, #males= 65; by strain- #AJ-F1= 19, #B6-F1= 26, #129-F1= 9, #NOD-F1= 8, #NZO-F1= 15, #CAST-F1= 9, #PWK-F1=3, #WSB-F1=13). Panel A- Total Cholesterol, B- plasma triglycerides, and C- Aortic lesions

**Figure S4. Plasma triglyceride chromosomal QTL graphs in three models of DO-F1 mice.**(A, B) Genome-wide plasma triglyceride QTLs on Chr 1 in female mice (A) and Chr 6 in a sex-additive model (B) at 24 weeks. Dashed lines correspond to P < 0.05 (significant), P < 0.1 (highly suggestive) or P < 0.63 (suggestive) thresholds. (C, D) Comparison of plasma triglyceride QTLs in three models on Chr 1 (C) and Chr 6 (D). Green, sex additive model; red, female mice; blue, male mice. Dashed lines correspond to P < 0.05 (significant) and P < 0.1 (highly suggestive). (E, F) The Best Linear Unbiased Predictors (BLUPs) coefficient plot of eight founder mice strains for plasma triglyceride QTL in females (E) and in a sex additive model (F) at 24 weeks. Color represents the eight founder mice strain as indicated.

**Figure S5. Allele frequencies of the 8 founder alleles across the chromosomes in DO-F1 mice.** The x-axis is the chromosome, and the y-axis is allele frequencies in DO-F1 mice. Each color denotes eight founder strains. The average allele frequency of each strain in all chromosomes except for the X chromosome is as follows. A/J: 11.69%, C57BL/6J: 13.50%, 129S1/SvlmJ: 12.94%, NOD/LtJ: 12.52%, NZO/HILtJ: 14.17%, CAST/EiJ: 11.71%, PWK/PhJ: 10.95%, WSB/EiJ: 12.41%

**Supplementary Table legends**

**Table S1**. Nutrient constituents in AIN76 and high-fat and high-cholesterol (HFHC) diet.

**Table S2**. Effects of sex and genetic backgrounds in cardiometabolic traits in DO founder strains-F1 mice at 24 weeks. n = 102 (37 females and 65 males).

**Table S3**. Spearman correlation analysis of plasma traits measured between pre-diet and post-diet time points in each sex. n = 472 (238 females and 234 males).

**Table S4**. Significant and suggestive QTL results for atherosclerotic traits in three models. n = 461 (235 females and 226 males).

**Table S5**. List of 51 genes in female plasma TG QTL on chromosome 1 and 93 genes in male plasma TG QTL on chromosome 6.

**Table S6**. Suggestive QTL results for change of plasma analytes between pre-diet and post-diet in three models. n = 461 (235 females and 226 males).

**Table S7**. Expressed 28 genes in female aortic lesion area QTL on chromosome 10 and 58 genes in male aortic lesion area QTL on chromosome 19 and their correlation with aortic lesion area. n = 162 (85 females and 77 males).

**Table S8**. RNA-seq alignment statistics in 162 DO-F1 mice.

**Table S9**. Significant liver cis-eQTL on chromosomes 10 and 19 and trans-eQTL results for transcripts in sex additive model and strain difference in regression coefficient of the association between each transcript and marker SNP. n = 162 (85 females and 77 males).

**Table S10**. Co-localization between significant aortic lesion area QTLs and liver *cis*-eQTLs, and a direct correlation between the mapped trait and transcript.

**Table S11**. Aortic lesion area QTL analysis by mediating candidate genes in each sex. n = 162 (85 females and 77 males).

**Table S12**. Significant KEGG pathway results for fifteen *Nr1h3* target genes.

**References**

1 Raghupathy, N. *et al.* Hierarchical analysis of RNA-seq reads improves the accuracy of allele-specific expression. *Bioinformatics* **34**, 2177-2184, doi:10.1093/bioinformatics/bty078 (2018).

2 Yang, J., Zaitlen, N. A., Goddard, M. E., Visscher, P. M. & Price, A. L. Advantages and pitfalls in the application of mixed-model association methods. *Nat Genet* **46**, 100-106, doi:10.1038/ng.2876 (2014).

3 Sen, S. & Churchill, G. A. A statistical framework for quantitative trait mapping. *Genetics* **159**, 371-387 (2001).

4 Doerge, R. W. & Churchill, G. A. Permutation tests for multiple loci affecting a quantitative character. *Genetics* **142**, 285-294 (1996).

5 Lander, E. & Kruglyak, L. Genetic dissection of complex traits- guidelines for interpreting and reporting linkage results. *Nat Genet* **11**, 241-247 (1995).
